# Is Vitamin D Fortification of Dairy Products Effective for Improving Vitamin D Status? A Systematic Review and Meta-Analysis of Randomised Controlled Trials

**DOI:** 10.3390/nu17233757

**Published:** 2025-11-29

**Authors:** Cheuk Lun Wong, D. Ian Givens, Anu M. Turpeinen, Xinyue Liu, Jing Guo

**Affiliations:** 1 School of Food Science and Nutrition, Faculty of Environment, University of Leeds, Leeds LS2 9JT, UK; fs23xl@leeds.ac.uk (X.L.); s.j.guo@leeds.ac.uk (J.G.); 2 Institute for Food, Nutrition and Health, University of Reading, Reading RG6 6EU, UK; d.i.givens@reading.ac.uk; 3 Valio Ltd., R&D, 00370 Helsinki, Finland; anu.turpeinen@valio.fi

**Keywords:** cheese, dairy, fortification, milk, vitamin D, vitamin D deficiency, yoghurt

## Abstract

**Background/objectives**: Given the suboptimal vitamin D intake and status among the United Kingdom population, the Scientific Advisory Committee on Nutrition is seeking suitable food vehicles for vitamin D fortification. Thus, this study aimed to examine the efficacy of vitamin D-fortified dairy products in improving serum 25-hydroxyvitamin D [25(OH)D] concentration using data from randomised controlled trials (RCTs). **Methods**: PubMed, Embase, and Web of Science were searched from inception until October 2024. Studies were included if they were RCTs with intervention groups administered vitamin D-fortified dairy products and control groups administered unfortified dairy products, as well as examining the effects on serum 25(OH)D concentration. **Results**: There were 35 RCTs eligible for inclusion, involving 4965 participants (intervention: 2526; control: 2439). The results showed that serum 25(OH)D concentrations were significantly increased by vitamin D_3_-fortified milk/milk powder (*n* = 15, mean difference (MD): 18.31 nmol/L, 95% confidence interval (CI): 13.30–23.33 nmol/L, *I*^2^ = 95%), vitamin D_3_-fortified yoghurt/yoghurt drinks (*n* = 11, MD: 26.22 nmol/L, 95% CI: 18.67–33.77 nmol/L, *I*^2^ = 97%), vitamin D_2_-fortified milk/milk powder (*n* = 3, MD: 11.61 nmol/L, 95% CI: 9.31–13.91 nmol/L, *I*^2^ = 0%), vitamin D-fortified (type not specified) milk/milk powder (*n* = 8, MD: 13.59 nmol/L, 95% CI: 8.54–18.64, *I*^2^ = 98%), and vitamin D-fortified (type not specified) yoghurt/yoghurt drinks (*n* = 4, MD: 27.74 nmol/L, 95% CI: 16.83–38.64 nmol/L, *I*^2^ = 91%), but insignificantly increased by vitamin D_3_-fortified cheese (*n* = 5, MD: 16.78 nmol/L, 95% CI: −3.61–37.16, *I*^2^ = 99%). However, the results of vitamin D_3_-fortified cheese became significant when leave-one-out analysis was performed by omitting one RCT (MD: 24.13 nmol/L, 95% CI: 4.69–43.58, *I*^2^ = 90%). **Conclusions**: These findings provide evidence that vitamin D-fortified dairy products have the potential to improve serum 25(OH)D concentrations in populations.

## 1. Introduction

In the United Kingdom (UK), the Scientific Advisory Committee on Nutrition (SACN) report in 2016 confirmed that serum 25-hydroxyvitamin D [25(OH)D] concentrations lower than 25 nmol/L were associated with a higher risk of vitamin D deficiency, which may lead to an increased risk of rickets in young children and osteomalacia in adults [1]. Apart from its relationship with bone health, observational studies also suggest the importance of vitamin D for non-musculoskeletal functions, for example, cardiometabolism, immunity [2], and insulin function [3], although the mechanism is not completely clear. Meanwhile, the SACN [1] also established a reference nutrient intake (RNI) for vitamin D of 10 μg/day for the general UK population aged 4 years and above, including pregnant and lactating women, and a safe intake of 8.5–10 μg/day for infants aged under 1 year throughout the year. Consuming this amount of vitamin D ensures that 97.5% of the population could achieve and maintain a serum 25(OH)D concentration higher than 25 nmol/L when sunshine exposure is limited. Despite having set the vitamin D RNI of 10 μg/day, the SACN [1] have acknowledged the difficulty of attaining the vitamin D RNI simply from natural food sources. In light of this, an SACN [4] report sought potential food vehicles for vitamin D fortification to increase vitamin D intake and improve vitamin D status in the UK population [4].

The UK National Diet and Nutrition Survey (NDNS) in 2020 and 2025 consistently confirmed low vitamin D intake and status in the UK population, especially in girls aged 11–18 years [5,6]. Substantial proportions of children (11–18 years) (23%) and adults (19–64 years) (18%) have serum 25(OH)D concentrations lower than 25 nmol/L, and have vitamin D intakes (2.1 µg/day for children aged 1.5–18 years and 2.7 µg/day for adults aged >18 years) significantly below the vitamin D RNI [6]. Moreover, the continuous decline in meat consumption in the UK [7] has made the UK population even more vulnerable to inadequate vitamin D intake and vitamin D deficiency, given that meat and meat products are the major food source of dietary vitamin D for adults (19–64 years) in the UK [5,6], contributing 25% of the daily recommended intake [6]. Therefore, there is an urgent need to improve vitamin D intake and status in the UK.

Dairy products, including milk, yoghurt, and cheese, may be suitable food vehicles for vitamin D fortification. Not only are they staple foods in the traditional UK diet, but they have also proven successful as vitamin D fortification vehicles in countries with vitamin D fortification policies, such as Finland, the United States, and Canada, to improve vitamin D intake and status [8]. Although the overall trends in household purchases of dairy foods and consumption of dairy products are declining [6,9], it is still worth considering vitamin D fortification of dairy products, given that consumers’ preferences also play an important role when considering suitable food vehicles for fortification. It is worth noting that a survey by Clark et al. [10] found that vitamin D-fortified cow’s milk may be more preferred by UK older adults, second only to breakfast cereals, which have been a part of the UK voluntary vitamin D fortification policy since 2006 [11].

Previous systematic reviews and meta-analyses (SRMAs) have explored the efficacy and effectiveness of vitamin D-fortified foods in improving serum 25(OH)D concentrations [12,13,14,15,16,17], focusing on various food matrices. Some of the previous SRMAs provided combined results regardless of food matrices [13,14,15,16], and Gasparri et al. [17] only focused on vitamin D-fortified yoghurts. Therefore, the aim of this SRMA was to examine the efficacy of solely vitamin D-fortified dairy products (i.e., milk, yoghurt, and cheese) in improving serum 25(OH)D concentrations. This would provide a crucial quantitative update by identifying and including a larger number of recent and relevant RCTs using vitamin D-fortified dairy products as interventions. This comprehensive collection allows for the efficacy results to be analysed with a high degree of specificity, focusing solely on dairy products (i.e., milk, yoghurt, and cheese), rather than combining results across various food matrices. Furthermore, in order to provide evidence that is more related to real-world settings, the production/fortification methods of the vitamin D-fortified dairy products in RCTs (e.g., provided by dairy food manufacturers) are also examined in subgroup analyses. This study may contribute to the evidence needed for the consideration of dairy products for vitamin D fortification in the UK.

## 2. Materials and Methods

This present SRMA was conducted in accordance with the Cochrane Handbook for Systematic Reviews of Interventions [18], and reported in accordance with the Preferred Reporting Items for Systematic Reviews and Meta-Analyses (PRISMA) [19]. The protocol was registered in the International Prospective Register of Systematic Reviews (PROSPERO) (registration number CRD420251157766). The PRISMA checklist is presented in the Supplementary Materials.

### 2.1. Literature Search

The literature search was conducted on PubMed, Embase, and Web of Science from inception to October 2024. This was supplemented by hand searching reference lists from relevant SRMAs to ensure comprehensive coverage. The overall search strategy and keywords for the electronic search are detailed in the Supplementary Materials. The search was focused on randomised controlled trials (RCTs) investigating vitamin D-fortified dairy products and the vitamin D status of participants. The keywords were developed based on five key concepts, including ‘vitamin D’, ‘fortified’, ‘dairy products’, ‘human’, and ‘RCTs’.

### 2.2. Eligibility Criteria

The inclusion criteria were developed based on the Participants, Interventions, Comparators, Outcomes, and Study design (PICOS) framework, as follows: (1) Population: general population with no restriction on ethnicity, age, sex, health status, etc.; (2) Intervention: consumption of vitamin D-fortified dairy products; (3) Comparators: unfortified dairy products as placebo for comparison; (4) Outcome: response measured in serum 25(OH)D concentrations; and (5) Study design: human RCTs. Furthermore, the language of studies was limited to English only.

The exclusion criteria for the studies included (1) duplicate, (2) studies were not human RCTs, (3) intervention group was not administered vitamin D-fortified dairy foods, (4) no placebo used or placebo group was not administered unfortified dairy foods, (5) serum 25(OH)D concentration at baseline or/and after intervention was not reported, (6) the form of vitamin D was not D_2_ or D_3_, (7) inclusion of other non-dairy vitamin D-fortified foods in the intervention group, and (8) serving size of the dairy products was not reported. Studies without full texts found were also excluded.

### 2.3. Study Selection

The study selection process was conducted independently by two researchers (C.L.W. and X.L.). After removing duplicates, the abstracts and titles of the studies were screened based on whether the studies were relevant to vitamin D-fortified dairy foods and human RCTs. Then, the full texts of the eligible studies were retrieved and screened based on the predefined eligibility criteria. Any disagreements regarding study eligibility were resolved by the reviewers through discussion and consensus.

### 2.4. Data Extraction and Synthesis

Key study information was extracted by C.L.W. and then independently verified by a second researcher (X.L.). Any discrepancies were discussed and resolved among the researchers. Information from the eligible studies was extracted including (1) study design, (2) number of participants in the intervention and placebo groups (3) health status, ethnicity, age, and body mass index (BMI) at baseline of the participants, (4) season that the trial was conducted, (5) trial duration, (6) forms of vitamin D and daily dose (µg/day) of vitamin D from the fortified dairy products, (7) serum 25(OH)D concentrations at baseline and endpoint in the intervention and placebo groups (8) the production/fortification methods and (9) vitamin D concentrations of the vitamin D-fortified dairy products. The unit of serum 25(OH)D concentration (1 ng/mL = 2.5 nmol/L) and vitamin D levels (40 IU = 1 μg) were presented in nmol/L and μg, respectively [20,21]. Unit conversions were made where necessary.

For serum 25(OH)D concentrations reported in the included RCTs, all were converted to be presented in arithmetic mean ± standard deviation (SD). Where necessary, SD was calculated based on 95% confidence interval (95% CI) or standard error of mean (SEM) using the formulae described in Black et al. [13] and the Cochrane Handbook [18], respectively. One of the studies [22] provided the geometric mean of serum 25(OH)D concentration and its arithmetic mean was estimated based on inequality [23], so the estimated arithmetic mean was considered lower than or equal to the geometric mean. The absolute mean differences (MDs) in serum 25(OH)D concentrations were calculated from baseline and endpoint in the intervention groups and control groups. Most studies did not provide the SDs of the changes in serum 25(OH)D concentrations; therefore estimates were made according to the Cochrane Handbook [18] regarding handling missing SDs for changes from baseline measurements.

### 2.5. Risk of Bias Assessment

The risk of bias of the included RCTs was assessed using the Cochrane risk-of-bias tool (RoB 2) [24] and was completed by two independent reviewers (C.L.W. and X.L.). Discrepancies were resolved by the two reviewers through discussion. There were five domains of the RCTs assessed, including ‘randomisation process’, ‘deviations from the intended interventions’, ‘missing outcome data’, ‘measurement of the outcome’, and ‘selection of the reported result’. Each domain could be graded in three levels of bias including ‘low risk’, ‘some concern’, and ‘high risk’.

### 2.6. Statistical Analysis

All statistical analyses were conducted using STATA 18.0 [25]. Restricted maximum likelihood meta-analysis was conducted to assess the efficacy of vitamin D-fortified dairy products on improving serum 25(OH)D concentration. The effect size was summarised as the mean difference (MD) in serum 25(OH)D concentration between intervention and control groups. The random effects model was chosen due to the high heterogeneity in the studies’ results. The heterogeneity of results between studies was assessed by the Cochrane Q test, and the level of inconsistency in the results of studies was presented in *I*^2^ [26]. *I*^2^ values of 25%, 50%, and 75%, indicated low, moderate, and high heterogeneity, respectively [26].

In order to examine the heterogeneity of the RCTs, subgroup analyses were conducted on age (child and teenager: <18 years; adult: ≥18 years), vitamin D status at baseline (Sufficient: >50 nmol/L; Insufficient: ≥25 and ≤50 nmol/L; Deficient: <25 nmol/L), body mass index (BMI) categories for adults (≥18 years) [27], dose of vitamin D consumed (<25 μg/day; ≥25μg/day), forms of vitamin D (vitamin D_2_; vitamin D_3_; not specified), types of dairy products (milk, yoghurt, and cheese), continent, processing/fortification methods of vitamin D-fortified dairy products, and risk of bias levels. There was one RCT [28] that used both vitamin D-fortified cheese and yoghurt as intervention groups. For easier data analysis, this was categorised as the group ‘yoghurt/yoghurt drink’, given that the participants’ compliance level for vitamin D-fortified yoghurt (96%) was higher than that of vitamin D-fortified cheese (84%).

Meta-regression analyses using a random effects model were performed to examine the heterogeneity between RCTs.

Leave-one-out analysis was also conducted to examine individual study effects on overall results.

Publication bias was assessed by performing Egger’s test for asymmetry [29], where *p* < 0.1 represented a significant publication bias. If publication bias was found, trim-and-fill analysis (estimator L0) would then be performed to examine potential missing studies and adjust the asymmetry of funnel plots [30]. Sensitivity analyses were also performed to examine publication bias.

### 2.7. Certainty of Evidence

The certainty of evidence was assessed using the Grades of Recommendation, Assessment, Development and Evaluation (GRADE) approach [31]. There were four classifications of the certainty of evidence in the GRADE approach, including ‘very low’, ’low’, ‘moderate’, and ‘high’. The initial rating of RCTs was at high confidence due to their rigorous methodology and study design (i.e., randomisation and control of confounding factors). However, the rating may be upgraded or downgraded based on the assessment of specific criteria, for example, risk of bias, inconsistency, indirectness, imprecision, and other considerations [31]. The assessment was conducted by two independent reviewers (C.L.W. and X.L.), and any disagreements were resolved through discussion.

## 3. Results

### 3.1. Study Selection

**Figure 1:**
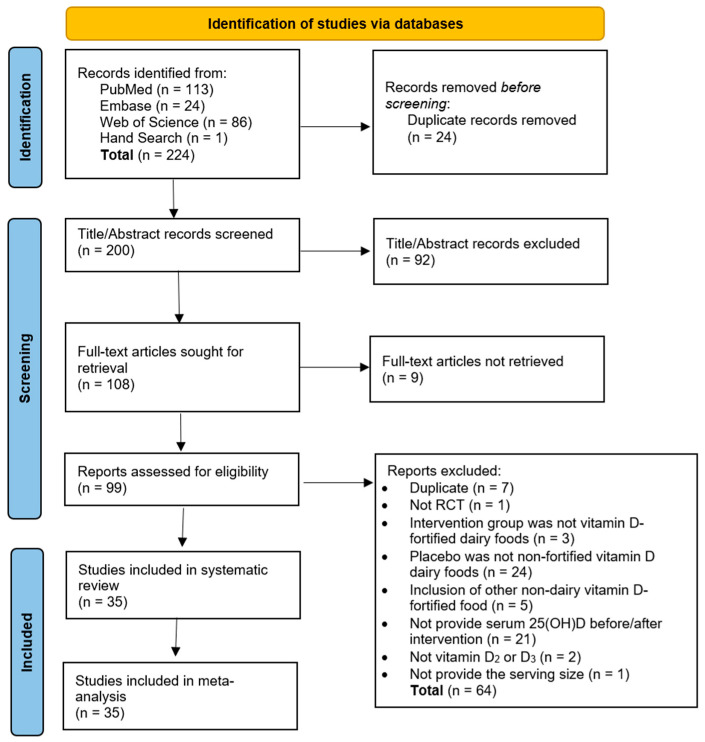
PRISMA flow diagram for study selection.

As shown in Figure 1, a total of 224 articles were identified from PubMed, Embase, Web of Science, and by hand searching, of which 24 were duplicates and excluded. Title and abstract screening was performed in 200 articles to identify relevant articles and 92 were excluded. The full texts of the remaining 108 articles were sought for retrieval and nine of them were not retrieved. Following the full text screening, 99 articles were examined and 64 of them did not meet the inclusion criteria and were excluded for various reasons, including ‘duplicate’ (*n* = 7), ‘not RCT’ (*n* = 1), ‘intervention group was not administered vitamin D-fortified dairy foods’ (*n* = 3), ‘placebo was not unfortified vitamin D dairy foods’ (*n* = 24), ‘inclusion of other non-dairy vitamin D-fortified food’ (*n* = 5), and ‘not provide serum 25(OH)D concentration before/after intervention’ (*n* = 21), ‘not vitamin D_2_ or D_3_’ (*n* = 2), and ‘not provide the serving size’ (*n* = 1). As a result, 35 articles were included in this SRMA.

### 3.2. Study Characteristics

The characteristics of the included 35 RCTs are described in Table 1. Approximately 60% of the RCTs were conducted in countries in Asia (*n* = 20) [32,33,34,35,36,37,38,39,40,41,42,43,44,45,46,47,48,49,50,51], followed by Europe (*n* = 9) [52,53,54,55,56,57,58,59,60], North America (*n* = 4) [28,61,62,63], and Oceania (*n* = 2) [22,64]. Moreover, only two RCTs were conducted in Southern Hemisphere countries [22,64], while the others were in Northern Hemisphere countries. While 10 RCTs did not report when the interventions were conducted [32,36,38,40,43,44,45,52,54,63], 23 RCTs were conducted in the darker phase of the year (e.g., autumn and winter) [28,33,34,35,37,39,41,42,46,47,48,49,50,51,53,55,56,57,58,59,60,61,62], one was in the brighter phase of the year [22], and one was across all seasons [64].

A total of 4965 participants (intervention: 2526, control: 2439) were included. The mean ages of participants ranged from 1 to 85.8 years old. Moreover, 10 RCTs were conducted on children (age < 18 years) with a mean age of 9.0 ± 3.3 years [28,32,36,37,42,46,54,57,60,64], of which one was conducted on infants (~1 year) [64]. The other 25 RCTs were conducted on adults with a mean age of 46.1 ± 16.5 years [22,33,34,35,38,39,40,41,43,44,45,47,48,49,50,51,52,53,55,56,58,59,61,62,63]. As for the ethnicity/race of the participants, 18 RCTs did not provide information on ethnicity/race [33,34,35,45,47,48,49,50,51,52,53,54,56,59,60,61,62,64], while the rest of the 17 RCTs reported ethnicities/races, including Chinese (*n* = 2) [32,41], Malaysian (*n* = 2) [40,44], Indian (*n* = 2) [36,46], Iranian (*n* = 4) [37,38,39,43], Mongol (*n* = 1) [42], and White/Caucasian (*n* = 3) [28,58,63]. Green et al. [22] involved multiple ethnicities in their study, including European, Asian, and Indian, while Fisk et al. [55] reported skin colour ratio (White/Non-white), indicating that the majority of the participants were white (Table 1). Similarly, Hower et al. [57] reported that only two participants in the control groups were dark skinned, while the majority of participants were light skinned (Table 1).

Regarding the health status of the participants (Table 1), 20 RCTs were conducted in generally healthy participants [22,28,32,36,37,40,42,44,46,53,54,55,56,57,58,59,60,61,62,64], two of which were cohorts of postmenopausal women [40,59]. Furthermore, one was conducted in participants with impaired fasting glucose and elevated glycated haemoglobin [63], one involved participants with fasting blood glucose levels > 7 mmol/L [38], and six had participants with type 2 diabetes mellitus (T2DM) [33,34,35,39,43,49], one of which was a group of postmenopausal women [39]. Moreover, one used female participants with gestational diabetes mellitus [41], two were participants with metabolic syndrome [45,48], three were participants with abdominal obesity [47,50,51], and one was heavily dependent long-stay elderly participants (mean age 84 years) [52].

In relation to the vitamin D-fortified dairy products in the RCTs (as shown in Table 1), vitamin D-fortified liquid milk (*n* = 13) [32,36,37,40,42,46,52,53,54,57,58,60,64], milk powder (*n* = 3) [22,44,55], yoghurt (*n* = 5) [38,39,45,48,56], yoghurt drink (*n* = 6) [33,34,35,41,43,49], cheese (*n* = 4) [59,61,62,63], milk and yoghurt (*n* = 3) [47,50,51], and cheese and yoghurt (*n* = 1) [28] were found as the intervention groups in the 35 RCTs. Regarding the types of vitamin D used in the interventions, of the 35 RCTs, 23 used vitamin D_3_ [22,28,32,33,34,35,36,38,39,41,45,47,50,51,52,53,56,58,59,61,62,63,64], one used vitamin D_2_ [46], one used both vitamin D_2_ and vitamin D_3_ [55], and 10 did not specify the type of vitamin D used in the interventions [37,40,42,43,44,48,49,54,57,60]. In addition, of the 35 included RCTs, 23 RCTs solely fortified with vitamin D [22,28,33,34,35,36,39,41,42,43,46,47,48,49,50,51,52,55,57,59,61,62,63], whereas 23 RCTs co-fortified vitamin D with minerals and/or other vitamins, including calcium (*n* = 7) [32,37,38,40,45,53,56], multiple vitamins (*n* = 1) [54], iron (*n* = 2) [58,64], multiple minerals (*n* = 1) [44], and both vitamins and minerals (*n* = 1) [60]. The dose of vitamin D consumed by the participants ranged from 2.5 µg/day to 100 µg/day, with a mean ± SD of 25.3 ± 23.9 µg/day. The 35 included RCTs had a median trial duration of 12 weeks (range: 4–104 weeks).

The production methods and vitamin D concentrations of the vitamin D-fortified dairy products are presented in Table S1. Of the 35 included RCTs, 14 RCTs did not provide the production method of the fortified dairy products [32,33,34,35,37,38,43,49,52,53,54,59,61,64], 15 RCTs mentioned that the fortified dairy products were provided by dairy product manufacturers [22,28,39,40,41,42,44,45,46,55,57,58,60,62,63], two RCTs reported that vitamin D was directly mixed with the dairy products [36,56], and four reported the use of nanoencapsulation of vitamin D for fortification [47,48,50,51]. Regarding the vitamin D concentrations, there were two RCTs [40,44] that did not report the serving size and therefore their vitamin D concentrations could not be reported in per 100 g or per 100 mL. Moreover, it could be observed that the vitamin D concentrations of vitamin D-fortified cheese (median: 132.35 µg/100 g, range: 9.5–333 µg/100 g) were generally higher than those of fortified milk (median: 1.375 µg/100 mL, range: 0.75–18.8 µg/100 mL), fortified milk powder (median: 19.2 µg/100 g, range: 6.7–20.8 µg/100 g), yoghurt (median: 25 µg/100 g, 5–325 µg/100 g), and yoghurt drink (median: 5 µg/100 mL, range: 4.03–25 µg/100 mL).

### 3.3. Risk of Bias Assessment

The results of the risk of bias assessment are reported in Figure S1. For the overall risk of bias of the 35 included RCTs, 10 of them were graded as ‘low risk’, and 23 and 2 were graded as ‘some concerns’ and ‘high risk’, respectively. Of the included RCTs, 21 RCTs did not provide study protocols or the study protocols could not be accessed online. Therefore, this contributed to ‘some concerns’ in domain 5, ‘selection of the reported result’. There were two RCTs graded as ‘high risk’ due to the bias in ‘deviations from the intended interventions’ and ‘randomisation process’.

### 3.4. Efficacy of Vitamin D-Fortified Dairy Products in Improving Serum 25(OH)D Concentration

#### 3.4.1. Vitamin D-Fortified Milk/Milk Powder

**Figure 2:**
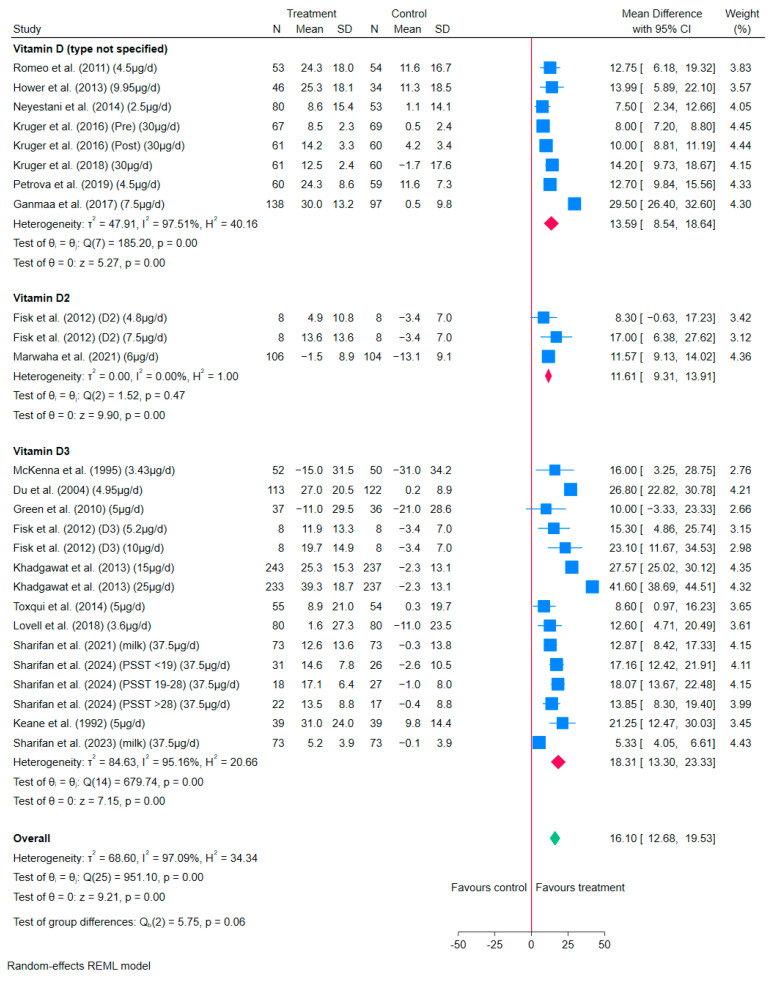
Forest plot of the effects of vitamin D-fortified milk/milk powder compared to control milk/milk powder on the changes in MD of serum 25(OH)D concentrations (nmol/L) in 19 RCTs with 26 data points [22,32,36,37,40,42,44,46,47,50,51,52,53,54,55,57,58,60,64]. The square represents the effect size of each study, and the diamond represents the pooled effect across studies. The width of each shape indicates its corresponding 95% CI.

The overall meta-analysis results showed that the consumption of vitamin D-fortified milk/milk powder resulted in statistically significant increased serum 25(OH)D concentrations, although the results were highly heterogeneous (MD: 16.10 nmol/L, 95% CI: 12.68–19.53, *I*^2^ = 97%) (Figure 2).

By the types of vitamin D used for fortification, consuming vitamin D_3_-fortified milk/milk powder showed a statistically significant increase in serum 25(OH)D concentration, although with high heterogeneity (MD: 18.31 nmol/L, 95% CI: 13.30–23.33 nmol/L, *I*^2^ = 95%). Although consuming vitamin D_2_-fortified milk/milk powder significantly increased serum 25(OH)D concentration (MD: 11.61 nmol/L, 95% CI: 9.31–13.91 nmol/L, *I*^2^ = 0%), it is worth noting that this meta-analysis was based on only two RCTs (three data points) (Figure 2). For RCTs that did not specify the type of vitamin D in their interventions, the meta-analysis results also suggested a statistically significant increase in serum 25(OH)D concentration by consuming vitamin D (not specified)-fortified milk/milk powder (MD: 13.59 nmol/L, 95% CI: 8.54–18.64 nmol/L, *I*^2^ = 98%), although the results were highly heterogeneous (Figure 2). In addition, there were no significant differences between vitamin D_2_, D_3_, and D (not specified) as fortificants on the effect of serum 25(OH)D concentration (*p* = 0.06).

The results of the subgroup analysis of vitamin D-fortified milk/milk powder are shown in Table S2. In general, the heterogeneity of the results remained high (*I*^2^ > 75%).

Regarding leave-one-out analysis, the meta-analysis results of vitamin D-fortified milk/milk powder (Figure S2) remained statistically significant with the effect of increasing serum 25(OH)D concentration.

#### 3.4.2. Vitamin D-Fortified Yoghurt and Yoghurt Drinks

**Figure 3:**
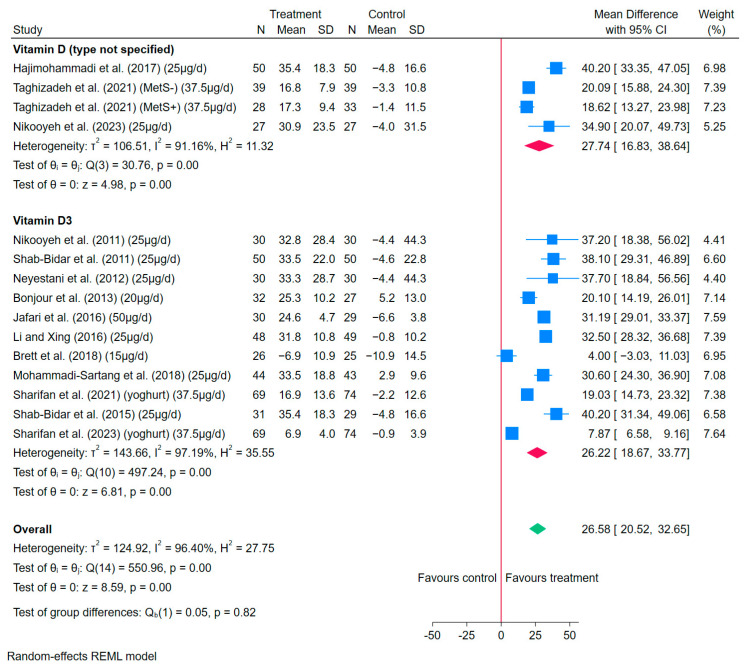
Forest plots of the effects of vitamin D-fortified yoghurt/yoghurt drinks compared to control yoghurt/yoghurt drinks on the changes in MD of serum 25(OH)D concentrations (nmol/L) in 14 RCTs with 15 data points [33,34,35,38,39,41,43,45,47,48,49,50,51,56]. The square represents the effect size of each study, and the diamond represents the pooled effect across studies. The width of each shape indicates its corresponding 95% CI.

The overall meta-analysis results showed that consuming vitamin D-fortified yoghurt/yoghurt drinks produced a statistically significant increase in serum 25(OH)D concentrations (MD: 26.58 nmol/L, 95% CI: 20.52–32.65, *I*^2^ = 96%) (Figure 3). However, there was a high heterogeneity among the results.

By the types of vitamin D for fortification, the meta-analysis results showed that consuming vitamin D_3_-fortified yoghurt/yoghurt drinks produced statistically significant increased serum 25(OH)D concentrations (MD: 26.22 nmol/L, 95% CI: 18.67–33.77 nmol/L, *I*^2^ = 97%), but also with high heterogeneity (Figure 3). For RCTs not specifying the type of vitamin D, the results also demonstrated statistically significant increases in serum 25(OH)D concentrations (MD: 27.74 nmol/L, 95% CI: 16.83–38.64 nmol/L, *I*^2^ = 91%), and the results were highly heterogeneous (Figure 3). Moreover, there were no significant differences between vitamin D_3_ and D (not specified) as fortificants in relation to the responses in serum 25(OH)D concentrations (*p* = 0.82).

The results of subgroup analysis are reported in Table S3. In general, the heterogeneity of the results remained high (*I*^2^ > 75%).

Regarding leave-one-out analysis, the meta-analysis results for vitamin D-fortified yoghurt/yoghurt drinks remained statistically significant with the effects of increasing serum 25(OH)D concentrations (Figure S3).

#### 3.4.3. Vitamin D-Fortified Cheese

**Figure 4:**
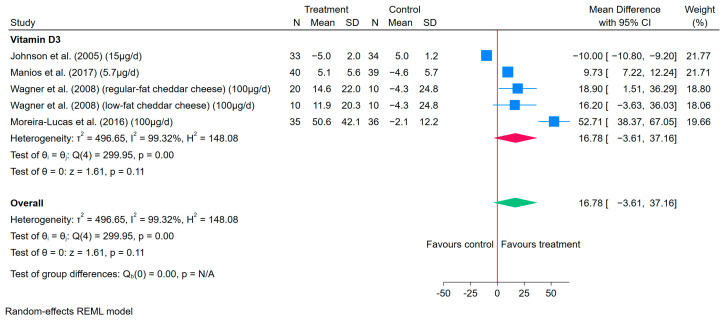
Forest plots of the effects of vitamin D-fortified cheese compared to control cheese on the changes in MD of serum 25(OH)D concentrations (nmol/L) in four RCTs with five data points [59,61,62,63]. The square represents the effect size of each study, and the diamond represents the pooled effect across studies. The width of each shape indicates its corresponding 95% CI.

The meta-analysis results showed that consuming vitamin D3-fortified cheese was not statistically significant in increasing serum 25(OH)D concentrations, with high heterogeneity (MD: 16.78 nmol/L, 95% CI: −3.61–37.16, *I*^2^ = 99%) (Figure 4).

However, when leave-one-out analysis was performed, with the exclusion of one RCT [61], the results became statistically significant, with an effect on increasing serum 25(OH)D concentrations (MD: 24.13 nmol/L, 95% CI: 4.69–43.58, *I*^2^ = 90%) (Figure S4), indicating the excluded RCT had a high influence on the meta-analysis results of vitamin D-fortified cheese.

The results of subgroup analysis are presented in Table S4. In general, the heterogeneity of the results remained high (*I*^2^ > 75%).

### 3.5. Meta-Regression

The meta-regression analyses surprisingly showed a non-significant association between the MD of serum 25(OH)D concentrations and serum 25(OH)D concentrations of participants at baseline (*p* = 0.074) (Figure S5) but a significant association with the BMI of participants at baseline (*p* < 0.001) (Figure S6).

### 3.6. Publication Bias

The results of Egger’s test for asymmetry and the trim-and-fill analysis are reported in Table S6. The funnel plots of the included 35 studies (Figure S7), the subgroups of the types of dairy products and vitamin D (Figure S8), and the subgroups of the types of dairy products (Figure S9) are also presented. As shown in Figure S7, there was an asymmetry in the funnel plot of the included 35 studies (i.e., vitamin D-fortified dairy products), which suggested a potential publication bias (*p* = 0.016). However, the trim-and-fill analysis did not impute any missing studies (Table S6). Therefore, the overall effect remained unchanged (MD: 19.34, 95% CI: 15.80–22.89). Furthermore, there was also a significant publication bias found in RCTs with vitamin D-fortified yoghurt/yoghurt drinks (*n* = 15, *p* = 0.045) (Table S6, Figure S9). The trim-and-fill analysis was able to impute three potential missing studies, with an adjusted result of MD: 23.36 nmol/L, 95% CI: 17.28–29.44 nmol/L (Table S6), which was originally MD: 26.58 nmol/L, 95% CI: 20.52–32.65 (Figure 3).

Additionally, the sensitivity analysis based on the number of participants (i.e., *n* ≥ 100 and *n* < 100) showed that RCTs with a smaller number of participants (*n* = 55 ± 24) had significant publication biases (*p* = 0.037) (Table S6) and asymmetries in the funnel plot (Figure S10). However, the trim-and-fill analysis was unable to impute any potential missing studies (Table S6). In contrast, there were no significant publication biases found in RCTs with participants ≥ 100 (*p* = 0.342) (Table S6). Given that a significant publication bias was found in RCTs involving vitamin D-fortified yoghurt/yoghurt drinks (*p* = 0.045), a sensitivity analysis with the exclusion of those RCTs was performed and the results showed that there was no significant publication bias found when excluding RCTs involving vitamin D-fortified yoghurt drinks (*p* = 0.162) (Table S6).

### 3.7. Certainty of Evidence

The overall certainty of evidence ranged from ‘very low’ to ‘high’ (Table S7). The certainty of evidence from the meta-analyses of vitamin D-fortified milk/milk powder (overall) and vitamin D_3_-fortified milk/milk powder was ’moderate’ and ‘high’, respectively. On the contrary, the meta-analysis of vitamin D (type not specified)-fortified milk/milk powder, vitamin D (type not specified)-fortified yoghurt/yoghurt drinks, and vitamin D_3_-fortified cheese resulted in ‘very low’ certainty of evidence due to very/extremely serious imprecision and inconsistency and serious risk of bias. Furthermore, the meta-analysis results of vitamin D2-fortified milk/milk powder, vitamin D-fortified yoghurt/yoghurt drinks (overall), and vitamin D3-fortified yoghurt/yoghurt drinks showed ‘low’ certainty of evidence.

## 4. Discussion

This present SRMA showed that the consumption of vitamin D-fortified milk/milk powder, yoghurt/yoghurt drinks and cheese are efficacious in improving serum 25(OH)D concentration, indicating it may be an effective strategy for tackling vitamin D deficiency, regardless of the form of vitamin D. The findings are supported by previous SRMAs which similarly showed that consuming vitamin D-fortified dairy products was effective in raising serum 25(OH)D concentrations [12,17]. While a previous SRMA found that vitamin D_3_ may be a more potent fortificant than vitamin D_2_ in improving serum 25(OH)D concentrations [14], our SRMA showed no significant difference between vitamin D_3_- and vitamin D_2_-fortified milk/milk powder in relation to the response of serum 25(OH)D concentration after consumption, which may be due to only two RCTs (three data points) using vitamin D_2_ in their intervention groups. In fact, there is already strong evidence showing that vitamin D_3_ is more efficacious than vitamin D_2_ in improving vitamin D status, which may be attributed to the chemical structures of their respective metabolites (i.e., 25(OH)D_2_ and 25(OH)D_3_), of which 25(OH)D_2_ has a lower affinity to vitamin D binding protein because of its extra methyl group [65]. In addition, it is also believed that dietary 25(OH)D_3_ may be even more effective in raising serum 25(OH)D concentration than vitamin D_3_ [66,67]. It is worth noting that apart from vitamin D_2_ and D_3_, the European Food Safety Authority (EFSA) also approved human consumption of 25(OH)D_3_ at up to 10 µg/day for children aged ≥ 11 and adults, including pregnant and lactating women [68], indicating that it may also be a potential fortificant. However, considering countries with vitamin D fortification policies such as Finland and the US, vitamin D_3_ may be a more common form of vitamin D for food fortification [4] and it is also cheaper than other forms of vitamin D.

In relation to the production/fortification methods of vitamin D-fortified dairy products, vitamin D fortification usually takes place during dairy food processing. Oil-based or water-dispersible vitamin D premix could be added directly to the milk during processing [69]. Oil-based vitamin D premix needs to be added to the milk stream after the separation of cream, while water-dispersible vitamin D premix could be added anywhere in the milk stream [69]. Therefore, vitamin D premix added to milk may go through the same raw milk processing steps, for example, standardisation, homogenisation, and heat treatment (e.g., pasteurisation, ultra-heat treatment (UHT)) [70,71]. Vitamin D-fortified milk could be further processed to produce vitamin D-fortified yoghurt and cheese with additional steps such as fermentation. Our SRMA also included subgroup analysis based on the production/fortification methods used for the vitamin D-fortified dairy products in the included RCTs, in which some vitamin D-fortified dairy products were provided by dairy product manufacturers and some were produced in the researchers’ laboratory, although the results showed high heterogeneity (*I*^2^ > 75%) regardless of the production/fortification methods. Those provided by dairy product manufacturers may show more relevance to real-world settings where dairy manufacturers would be responsible for vitamin D fortification and dairy product production.

Similar to previous SRMAs [12,13,14,15,16,17], our SRMA results unsurprisingly showed high heterogeneity. This could be attributed to study variability, including but not limited to populations/ethnicities, locations, vitamin D doses, and durations of trials. Furthermore, individual variability may also contribute to the heterogeneity; for example, variability in BMI, baseline serum 25(OH)D concentration, and age [72]. Our subgroup analyses were conducted within each type of dairy product, including milk/milk powder, yoghurt/yoghurt drink, and cheese, and the heterogeneity was slightly reduced by the production/fortification methods of the vitamin D-fortified dairy products and continents, although the heterogeneity was still high. In addition, our meta-regression analyses revealed that BMI for adults at baseline was positively associated with the MD of serum 25(OH)D concentration (*p* < 0.001), which may be explained by BMI being negatively associated with serum 25(OH)D concentration [73,74], indicating a potentially lower baseline of serum 25(OH)D in participants with higher BMIs. Therefore, the consumption of vitamin D-fortified dairy products would lead to a greater increase in serum 25(OH)D concentration in participants with higher BMIs due to the lower baseline serum 25(OH)D concentration of those participants. It is believed that the inverse association between BMI and serum 25(OH)D may be due to less sunlight exposure caused by physical inactivity and vitamin D sequestration in adipose tissue, given that the skin’s capacity for the production of vitamin D may not be affected by BMI [75]. Moreover, a review by Mazahery and von Hurst [72] summarised factors that affect the response in serum 25(OH)D concentration in relation to vitamin D supplementation, and there was good evidence suggesting that higher BMI or body fat percentage was associated with smaller increases in serum 25(OH)D concentration. This would imply that higher doses of vitamin D may be needed for individuals with higher BMIs in order to achieve a similar response in serum 25(OH)D concentration to those with normal BMIs. Of course, reducing BMI may help release vitamin D from adipose tissue into the bloodstream and increase serum 25(OH)D concentration [76]. Regarding the relationship between baseline serum 25(OH)D concentration and the MD of serum 25(OH)D concentration, our SRMA failed to show a significant result (*p* = 0.074, Figure S5), which may be attributed to the high levels of heterogeneity between the included RCTs. However, a downward trend in the MD of serum 25(OH)D concentration is still observable, as baseline serum 25(OH)D concentrations increased. A previous SRMA by Nikooyeh et al. [16] revealed a quadratic dose–response relationship between serum 25(OH)D at baseline and responses in serum 25(OH)D, where higher responses can be observed in subjects with lower baseline serum 25(OH)D concentrations.

It is worth noting that the vitamin D doses (median: 25 μg/day; range: 2.5–100 μg/day) from vitamin D-fortified dairy products consumed by the participants in the included RCTs are generally higher than the vitamin D RNI of 10 μg/day, which would obviously lead to greater responses in serum 25(OH)D concentrations. Moreover, countries with vitamin D fortification of dairy products policies may have limitations on the vitamin D concentrations in dairy products. For example, the US permits voluntary vitamin D fortification of fluid milk and yoghurt with vitamin D concentrations that must not exceed 2.1 μg/100 g and 2.23 μg/100 g, respectively [77]. In addition, the vitamin D intake from vitamin D-fortified dairy products also depends significantly on the amount of consumption of such products. Therefore, modelling studies would be important for simulating the effects of vitamin D fortification of dairy products on vitamin D intake and responses in serum 25(OH)D concentration, as the data on population intake of dairy products would also be taken into account and potentially provide results that are more relevant to real-world settings. In fact, the SACN report in 2024 concluded that modelling studies would be required to identify suitable fortification vehicles and assess safe levels of fortification, with current vitamin D levels in fortified foods and supplement use taken into account [4]. The consideration of vitamin D intake from fortified foods and supplements in modelling studies using population data, such as NDNS, would help to prevent potential safety issues with vitamin D; for example, vitamin D intoxication due to vitamin D overdose. However, this health condition is rare given that a suggested hallmark of vitamin D intoxication is to achieve a serum 25(OH)D concentration of >375 nmol/L [78,79], implying a consumption of very high doses of vitamin D (e.g., >1250 µg/day) [80], which are hundreds of times higher than the UK vitamin D RNI (10 µg/day) [1] and 12.5 times more than the vitamin D upper limit (100 µg/day) established by the EFSA [81] and the Institute of Medicine (IOM) [82]. Although there are a few modelling studies regarding vitamin D fortification of milk in the UK [83,84], none of them have examined a wide variety of dairy products. It has been found that countries with vitamin D fortification of a wide variety of dairy products have demonstrated significantly greater vitamin D intakes compared to those with partial coverage or without fortification policies [85].

Consumer preferences are important factors when considering suitable food vehicles for fortification. Various factors could influence consumers’ choices of foods; for example, taste and texture, price, and the general perception of foods [86]. In a survey study with five groups of 120 older adults (>65 years) who were at risk of vitamin D deficiency [10], cow’s milk (58%) was the second-most preferred food product for vitamin D fortification, while breakfast cereals (64%) were the most preferred food products. However, breakfast cereals have already been voluntarily fortified with vitamin D since 2006 [11]. It is crucial that food vehicles for fortification, such as dairy products, are popularly and preferably consumed by the general UK population, as well as those who are at risk of vitamin D deficiency. However, it is important to highlight that the dietary intake of dairy products differs across age groups [5,6], with 11–18 years and 19–64 years having the lowest dairy intakes of 161 g/day and 144 g/day, respectively, according to the NDNS in 2025 [6]. Therefore, as mentioned above, it would be crucial to have modelling studies to simulate the appropriate vitamin D concentration for fortification so that it helps consumers to attain the UK vitamin D RNI of 10 µg/day. As another option, strategies to encourage at-risk groups, for example, adolescent girls, older adults, and ethnic minorities, to consume more dairy products may also be considered. In fact, the British Nutrition Foundation [87] recommends a daily consumption of 200 mL of whole/semi-skimmed/skimmed milk and 120 g of plain/low-fat yoghurt to the general UK population.

Regarding the strengths of our SRMA, our study was able to identify and include a larger number of RCTs of vitamin D fortification of dairy products compared to previous SRMAs where vitamin D-fortified dairy products were also reported [12,13,14,15,16,17], and was able to perform our meta-analyses based on the types of dairy products rather than combined results. Moreover, our SRMA also examined the production/fortification methods of the vitamin D-fortified dairy products and their efficacy in improving serum 25(OH)D concentrations, with the intention of showing relevance to real-world settings in which dairy manufacturers are normally responsible for dairy foods processing and vitamin D fortification. However, this would assume that the vitamin D-fortified dairy products provided by dairy product manufacturers in RCTs also underwent similar dairy food processing and vitamin D fortification procedures to those in real-world settings, rather than simply adding and mixing vitamin D to already processed dairy products. Comprehensive analyses, including trim-and-fill analysis and sensitivity analysis, were performed to examine publication biases, and our sensitivity analyses revealed a significant publication bias (*p* = 0.037) among RCTs with smaller numbers of participants (*n* < 100), which may imply that RCTs with smaller numbers of participants tend to publish larger positive effects. This information may be important for future SRMAs. Where possible, it may be beneficial for future SRMAs to focus on larger RCTs (e.g., *n* ≥ 100) so as to minimise publication bias.

Despite the clear strengths of our SRMA, there are limitations to our study. Given that only studies published in English were eligible for inclusion, this may potentially exclude some relevant studies published in other languages. Furthermore, our meta-analyses’ results were highly heterogeneous, despite multiple subgroup analyses being performed. Individual variability, for example, in age and metabolism, as well as inconsistencies in the measurement of serum 25(OH)D used in RCTs, also contributed to heterogeneity. It is also worth noting that parts of the included RCTs did not report the form of vitamin D or the fortification methods of vitamin D-fortified dairy products. Therefore, our subgroup analyses based on the form of vitamin D and fortification methods may have missed some relevant RCTs. It is recommended that the form of vitamin D and the fortification methods of dairy products should be clearly reported in future RCTs. Moreover, our SRMA was unable to draw a solid conclusion regarding the efficacy of vitamin D-fortified cheese in improving vitamin D status, given that the leave-one-out analysis of vitamin D-fortified cheese showed that one RCT had a substantial influence on the overall results. Apart from that, there were only four RCTs, with five data points, that used vitamin D-fortified cheese as an intervention, which is considerably fewer than the number of RCTs regarding vitamin D-fortified milk/milk powder and yoghurt/yoghurt drinks, indicating the need for more evidence from vitamin D-fortified cheese in relation to the response of serum 25(OH)D concentrations. In addition, our SRMA reported a significant risk of publication bias in the RCTs with vitamin D-fortified yoghurt/yoghurt drink interventions (Table S6 and Figure S9) and vitamin D-fortified dairy products (i.e., all included RCTs) (Table S6 and Figure S7). However, the trim-and-fill analysis could not impute any potential missing studies regarding the overall combined effect (i.e., all included RCTs) on serum 25(OH)D concentration, which may be due to the substantial heterogeneity between the included RCTs, indicating a potential overestimation of the response in serum 25(OH)D concentrations, and therefore, the overall results should be interpreted with caution.

## 5. Conclusions

This SRMA provides updated and additional evidence that consuming vitamin D-fortified dairy products, including milk/milk powder and yoghurt/yoghurt drinks, may be efficacious in increasing serum 25(OH)D concentration, which may be useful for policymakers when considering suitable potential vitamin D fortification vehicles. However, more evidence is needed to confirm the efficacy of vitamin D-fortified cheese in improving vitamin D status. Further modelling studies to simulate the effect of vitamin D fortification of a wide variety of dairy products on vitamin D intake and response in serum 25(OH)D concentration are essential.

## Figures and Tables

**Table 1:** Study characteristics of the included 35 RCTs.

Author (Year)	Study Design			Characteristics of Participants				Intervention Group			Placebo Group		
	Country	Int (*n*); Ctrl (*n*)	Trial Duration; Season	Health Status	Age (Years)	BMI at Baseline (kg/m^2^)	Ethnicity	Dairy Type; Vit D (µg/d)	Serum 25(OH)D at Baseline (nmol/L)	Serum 25(OH)D at Endpoint (nmol/L)	Placebo	Serum 25(OH)D at Baseline (nmol/L)	Serum 25(OH)D at Endpoint (nmol/L)
McKenna et al. (1995) [53]	Ireland	52; 50	6 months; Winter	Healthy	Median: 22.6, range: 17–54	NA	NA	Vit D_3_-fortified milk (Ca: 1525 mg/L); 3.4	77 ± 35	62 ± 26	Unfortified milk;	85 ± 39	54 ± 25
Du et al. (2004) [32]	China	113; 122	2 years; NA	Healthy	10	Int: 16.8 ± 2.6 Ctrl: 16.8 ± 2.6	Chinese	Vit D_3_-fortified milk (with Ca); 5.0	20.6 ± 8.8	47.6 ± 23.4	Unfortified milk;	17.7 ± 8.7	17.9 ± 9.0
Green et al. (2010) [22]	New Zealand	37; 36	12 weeks; Summer to Autumn	Healthy	Int: 28.0 (95% CI: 25.5–30.6) Ctrl: 28.8 (95% CI: 26.3–31.3)	Int: 23.3 (95% CI: 22.9–25.8) Ctrl: 23.7 (95% CI: 22.4–25.0)	European: Int (78.4%) Ctrl (91.7%); Asian: Int (16.2%) Ctrl (5.6%); Indian: Int (5.4%) Ctrl (2.8%)	Vit D_3_-fortified milk powder, dissolved in 200 mL of water; 5	≤76 ± 32.6 ^1^	≤65 ± 24.8 ^1^	Unfortified milk;	≤74 ± 30.6 ^1^	≤53 ± 26.0 ^1^
Romeo et al. (2011) [54]	Spain	53; 54	5 months; NA	Healthy	Boys: 11.4 ± 2.17; Girls: 11.5 ± 2.30	Int: 19.3 ± 3.88; Ctrl: 20.29 ± 3.30	NA	Vit D-fortified milk *; 4.5	68.98 ± 15.13	93.33 ± 19.85	Standard whole milk	68.7 ± 13.58	80.3 ± 18.65
Fisk et al. (2012) [55]	United Kingdom	D_2_ (5 µg): 8 D_3_ (5 µg): 8 D_2_ (10 µg): 8 D_3_ (10 µg): 8; Ctrl: 8	4 weeks; Winter	Healthy	Median (range): Int: 3.8 (2.0–6.8) Ctrl: 3.7 (2.0–6.2)	Median (range): Int: 15.6 (13.0–22.1) Ctrl: 15.4 (14.1–20.0)	Light skinned: Int (*n* = 46) Ctrl (*n* = 32); Dark skinned: Int (*n* = 0) Ctrl (*n* = 2)	Vit D_2_/D_3_-fortified malted milk drink; Vit D_2_: 4.8 and 7.5 Vit D_3_: 5.2 and 10	D_2_ (5 µg): 48.0 ± 26.6 ^3^ D_3_ (5 µg): 31.3 ± 22.1 ^3^ D_2_ (10 µg): 41.9 ± 14.1 ^3^ D_3_ (10 µg): 30.9 ± 29.1 ^3^	D_2_ (5 µg): 52.9 ± 20.53 ^4^ D_3_ (5 µg): 43.2 ± 15.924 * D_2_ (10 µg): 55.5 ± 10.75 ^4^ D_3_ (10 µg): 50.6 ± 21.48 ^4^	Unfortified malted milk drink;	33.5 ± 13.3 ^3^	30.1 ± 9.78 ^4^
Hower et al. (2013) [57]	Germany	46; 34	9 months; Winter to summer	Healthy	Median (range): Int: 3.8 (2.0–6.8) Ctrl: 3.7 (2.0–6.2)	Median (range): Int: 15.6 (13.0–22.1) Ctrl: 15.4 (14.1–20.0)	Light skinned: Int: *n* = 46 Ctrl: *n* = 32 Dark skinned: Int: *n* = 0 Ctrl: *n* = 2	Vit D-fortified growing-up milk; 9.975	60.23 ± 20.55 ^2^	71.63 ± 13.58 ^2^	Unfortified semi-skimmed cow’s milk	58.30 ± 21.2 ^2^	69.61 ± 13.08 ^2^
Khadgawat et al. (2013) [36]	India	15 µg: 243 25 µg: 233; 237	12 weeks; NA	Healthy	15 µg: 11.75 ± 1.08 25 µg: 11.75 ± 1.14 Ctrl: 11.74 ± 1.05	15 µg: 18.84 ± 3.66 25 µg: 18.62 ± 3.50 Ctrl: 18.94 ± 3.33	Indian	Vit D-fortified milk; 15 and 25	15 µg: 28.55 ± 13.1 25 µg: 29.85 ± 14.05	15 µg: 57.18 ± 16.88 25 µg: 69.18 ± 21.18	Unfortified milk; NA	29.35 ± 13.08	27.08 ± 13.1
Neyestani et al. (2014) [37]	Iran	80; 53	12 weeks; Winter	Healthy	10–12	Mean (SEM): Int: 18.3 (0.4) Ctrl: 18.3 (0.4)	Iranian	Vit D-fortified milk (with 500 mg of Ca); 2.5	25(OH)D_3_: 24.9 ± 12.5 ^5^	25(OH)D_3_: 33.5 ± 16.1 ^5^	Plain milk (with 240 mg/200 mL of calcium); non-detectable	25(OH)D3: 27.4 ± 13.8 ^5^	25(OH)D3: 28.5 ± 13.1 ^5^
Toxqui et al. (2014) [58]	Spain	55; 54	16 weeks; Winter	Healthy	Fe + vit D: 24.7 ± 4.6 Ctrl: 24.8 ± 4.1	Fe + vit D: 21.4 ± 3.0 Ctrl: 21.9 ± 3.0	Caucasian	Vit D_3_-fortified skimmed flavoured milk fortified (with 15 mg of Fe); 5	62.3 ± 20.8	71.2 ± 21.1	Skimmed flavoured milk with fortified iron (15 mg/500 mL)	62.9 ± 20.8	63.2 ± 18.3
Kruger et al. (2016) [40]	Malaysia	Pre: 67 Post: 61; Pre: 69 Post: 60	12 weeks; NA	Postmenopausal and premenopausal	Pre: Int 42 ± 5.1 Ctrl 41 ± 5.1 Post: Int 60 ± 3.9 Ctrl 59 ± 4.3	Pre: Int 23.0 ± 3.28 Ctrl 22.3 ± 3.21 Post: Int 23.4 ± 2.85 Ctrl 24.4 ± 2.88	Malaysian Chinese	Pre: Vit D-fortified milk (1000 mg of Ca) Post: Vit D-fortified milk (1200 mg of Ca); 30	Pre: 54.6 ± 2.49 Post: 57.3 ± 3.54	Pre: 63.1 ± 2.03 Post: 71.5 ± 2.98	Regular milk with 500 mg of calcium	Pre: 52.0 ± 2.72 Post: 59.3 ± 3.66	Pre: 52.5 ± 2.00 Post: 63.5 ± 3.03
Kruger et al. (2018) [44]	Malaysia	61; 60	12 months; NA	Healthy	Int: 59 ± 3.9 Ctrl: 60 ± 4.3	Int: 23.4 ± 2.94 Ctrl: 24.5 ± 2.94	Malaysian Chinese	Vit D-fortified milk powder (1200 mg of calcium, 96 mg of magnesium, 2.4 mg of zinc, and 4 g of FOS-inulin); 30	25(OH)D_3_: 62.3 ± 1.89	25(OH)D_3_: 74.8 ± 2.74	Regular milk	25(OH)D_3_: 64.8 ± 18.89	25(OH)D_3_: 63.1 ± 2.87
Lovell et al. (2018) [64]	New Zealand, Australia	80; 80	12 months; All season	Healthy	1 year ± 2 week	NA	NA	Vit D_3_-fortified growing-up milk-lite (fortified with 1.7 mg/100 mL of iron, probiotics and prebiotics); 3.6	90.4 ± 28.8	92.0 ± 25.5	Unfortified cow milk	85.9 ± 23.4	74.9 ± 23.5
Petrova et al. (2019) [60]	Spain	60; 59	5 months; winter	Healthy	11 ± 2.14	BMI (square root transformation) in mean (SE): Int: 4.34 (0.06) Ctrl: 4.51 (0.06)	NA	Vit D-fortified milk beverage (with vit A, B complex, C, and E, Ca, P, Zn, and fish oils) 4.5	66.0 ± 9.37 ^5^	90.3 ± 7.44 ^5^	Regular full milk	67.9 ± 7.30 ^5^	79.5 ± 7.30 ^5^
Marwaha et al. (2021) [46]	India	106; 104	3 months; winter	Healthy	Int: 10.3 ± 0.5 Ctrl: 10.4 ± 0.8	Int: 16.7 ± 3.3 Ctrl: 16.8 ± 3.2	Indian	Vit D_2_-fortified milk; 6	28.55 ± 9.075	27.025 ± 8.75	Unfortified milk	29.925 ± 9.475	16.825 ± 8.75
Sharifan et al. (2021) [47]	Iran	Milk: 73 Yoghurt: 69; Milk: 73 Yoghurt: 74	10 weeks; winter	Abdominal obesity	Int: Milk 40.42 ± 8.03 Yoghurt: 43.47 ± 7.21; Ctrl: Milk 40.26 ± 8.27 Yoghurt: 43.19 ± 7.21	Int: Milk 22.95 ± 2.96 Yoghurt: 23.8 ± 3.65 Ctrl: Milk 23.11 ± 3.16 Yoghurt: 23.27 ± 3.27	NA	Vit D_3_-fortified low-fat milk and Vit D_3_-fortified low-fat yoghurt; Milk: 37.5 Yoghurt: 37.5	Milk: 35.2 ± 12.875 Yoghurt: 35.35 ± 12.6	Milk: 47.75 ± 14.225 Yoghurt: 52.2 ± 14.4	Unfortified low-fat milk and yoghurt	Milk: 35.05 ± 12.9 Yoghurt: 38.35 ± 14.2	Milk: 34.725 ± 14.625 Yoghurt: 36.175 ± 9.825
Nikooyeh et al. (2011) [33]	Iran	30; 30	12 weeks; autumn and winter	T2DM patient	50.7 ± 6.1	Int: 29.2 ± 4.4 Ctrl: 29.9 ± 4.7	NA	Vit D_3_-fortified yoghurt drink; 25	25(OH)D_3_: 44.4 ± 28.7	25(OH)D_3_: 77.7 ± 28.6	Plain yoghurt	25(OH)D_3_: 41.6 ± 44.5	25(OH)D_3_: 37.2 ± 44
Wagner et al. (2008) [62]	Canada	DC: 20 DLF: 10; 10	8 weeks; winter	Healthy	DC: 28.7 ± 11.4 DLF: 30.6 ± 11.7	DC: 25.2 ± 5.0 DLF: 24.2 ± 3.3	NA	DC: Vit D_3_-fortified regular-fat cheddar cheese DLF: Vit D_3_-fortified low-fat cheddar cheese; DC: 100 DLF: 100	DC: 50.7 ± 18.9 DLF: 57.5 ± 18.4	DC: 65.3 ± 24.1 DLF: 69.4 ± 21.7	Unfortified cheddar cheese	55.0 ± 25.3	50.7 ± 24.2
Taghizadeh et al. (2021) [48]	Iran	MetS−: 39 MetS+: 28; MetS−: 39 MetS+: 33	10 weeks; winter	With MetS and without MetS	MetS−: 9.67 ± 7.15 MetS+: 1.6 ± 8.2	MetS−: 2.87 ± 3.12 MetS+: 4.72 ± 3.25	NA	Vit D-fortified yoghurt; 37.5	MetS−: 34.925 ± 10.675 MetS+: 36.175 ± 15.175	MetS−: 51.75 ± 12.925 MetS+: 53.475 ± 16.35	Plain yoghurt	MetS−: 38.75 ± 14.175 MetS+: 38.125 ± 14.55	MetS−: 35.5 ± 9.975 MetS+: 36.75 ± 9.825
Ganmaa et al. (2017) [42]	Mongolia	138; 97	7 weeks; winter	Healthy	Int: 10.0 ± 0.8 Ctrl: 9.7 ± 0.9	Int: 16.4 ± 1.9 Ctrl: 16.3 ± 1.8	Mongol	Vit D-fortified cow’s milk; 7.52	19.25 ± 10	49.25 ± 15.25	Unfortified cow’s milk	18 ± 9.5	18.75 ± 9
Sharifan et al. (2024) [51]	Iran	PSST score <19: 31 19–28:18 >28: 22; PSST score <19: 26 19–28:27 >28: 27	10 weeks; winter	Abdominal obesity	Int: PSST <19: 43.26 ± 7.94 19–28: 41.52 ± 8.15 >28: 38.75 ± 5.37 Ctrl: PSST <19: 42.34 ± 7.13 19–28: 41.33 ± 9.22 >28: 39.01 ± 4.44	NA	NA	Vit D_3_-fortified low-fat milk and vit D_3_-fortified low-fat yoghurt; Milk: 37.5 Yoghurt: 37.5	PSST score: <19: 35.25 ± 14.875 19–28: 35.8 ± 11.3 >28: 39.025 ± 11.7	PSST score: <19: 49.85 ± 16.525 19–28: 52.9 ± 13.075 >28: 52.525 ± 11.45	Unfortified low-fat milk and yoghurt	PSST score: <19: 42.825 ± 12 19–28: 41.875 ± 11.375 >28: 35.85 ± 15.125	PSST score: <19: 40.25 ± 11.1 19–28: 40.85 ± 11.75 >28: 35.5 ± 16.7
Johnson et al. (2005) [61]	US	35; 37	2 months; Winter	Healthy	≥60	NA	NA	Vit D_3_-fortified process cheese; 15	57.5 ± 3.5	52.5 ± 3.5	Unfortified cheese	50 ± 3.0	55 ± 2.75
Shab-Bidar et al. (2011) [34]	Iran	50; 50	12 weeks; Autumn to winter	T2DM patient	52.5 ± 7.4	Int: 28.6 ± 4.0 Ctrl: 30.0 ± 4.2	NA	Vit D_3_-fortified doogh **; 25	38.5 ± 20.2	72.0 ± 23.5	Plain doogh **	38.0 ± 22.8	33.4 ± 22.8
Neyestani et al. (2012) [35]	Iran	30; 30	12 weeks; autumn and winter	T2DM patient	Int: 51.5 ± 5.4 Ctrl: 50.8 ± 6.7	Int: 29.2 ± 4.4 Ctrl: 29.9 ± 4.7	NA	Vit D_3_-fortified doogh **; 25	44.4 ± 28.7	77.7 ± 28.6	Plain doogh **	41.6 ± 44.5	37.2 ± 44
Bonjour et al. (2013) [56]	France	32; 27	56 days; Winter	Healthy	Int: 85.8 ± 1.2 Ctrl: 85.1 ± 1.3	Int: 26.2 ± 0.7 Ctrl: 26.6 ± 1.0	NA	Vit D_3_-fortified yoghurt (with 800 mg of Ca); 20	19.2 ± 1.2	39.5 ± 3.3	Yoghurt (fortified with 280 mg of calcium only)	16.2 ± 0.6	21.4 ± 2.7
Jafari et al. (2016) [39]	Iran	30; 29	12 weeks; late autumn to winter	T2DM patient, postmenopausal	Int: 57.8 ± 5.5 Ctrl: 56.8 ± 5.7	Int: 28.00 ± 0.82 Ctrl: 29.30 ± 0.72	Iranian	Vit D-fortified yoghurt; 50	62.23 ± 4.52	86.83 ± 4.87	Plain yoghurt	62.72 ± 4.27	56.13 ± 2.89
Li and Xing (2016) [41]	China	48; 49	16 weeks; Winter	Women with GDM	24–32	Int: 24.61 Ctrl: 25.5	Chinese	Vit D_3_-fortified yoghurt; 25	42 ± 11.5	73.75 ± 14.23	Plain yoghurt	40.5 ± 8.5	39.75 ± 11.25
Hajimohammadi et al. (2017) [43]	Iran	50; 50	12 weeks; NA	T2DM patient	Int: 52.4 ± 8.4 Ctrl: 52.6 ± 6.3	Int: 28.6 ± 4.0 Ctrl: 30.0 ± 4.2	Iranian	Vit D-fortified yoghurt drink; 25	38.5 ± 20.2	72.0 ± 23.5	Unfortified yoghurt drink	38.0 ± 22.8	33.4 ± 22.8
Manios et al. (2017) [59]	Greece	40; 39	8 weeks; Winter	Postmenopausal women	Int: 62.6 ± 6.0 Ctrl: 63.2 ± 5.9	Int: 28.0 ± 3.8 Ctrl: 29.0 ± 2.9	NA	Vit D_3_-fortified Gouda-type cheese (fat reduced); 5.7	47.3 ± 15.2	52.5 ± 12.0	Unfortified, fat-reduced Gouda-type cheese	42.9 ± 17.7	38.3 ± 18.9
Brett et al. (2018) [28]	Canada	26; 25	6 months; autumn to spring	Healthy	Int: 5.0 ± 1.8 Ctrl: 5.4 ± 2.0	z score: Int: 0.55 ± 0.98 Ctrl: 0.81 ± 0.88	White: Int: *n* = 18 Ctrl: *n* = 13	Vit D_3_-fortified cheddar cheese and Vit D_3_-fortified yoghurt; 15	25(OH)D_3_: 65.3 ± 12.2	25(OH)D_3_: At 3 months, 64.7 ± 12.2 At 6 months: 58.4 ± 8.7	Unfortified cheddar cheese or drinkable yoghurts	25(OH)D_3_: 67.5 ± 15.1	25(OH)D_3_: At 3 months: 58.3 ± 15.3 At 6 months: 56.6 ± 13.9
Mohammadi-Sartang et al. (2018) [45]	Iran	44; 43	10 weeks; NA	With MetS	Int: 45.4 ± 8.9 Ctrl: 45.6 ± 8.7	Int: 30.1 ± 2.6 Ctrl: 30.8 ± 2.2	NA	Vit D_3_-fortified yoghurt (with calcium and inulin); 25	65.1 ± 34.9	98.6 ± 28.8	Plain yoghurt	65.2 ± 32.6	70.2 ± 32.6
Shab-Bidar et al. (2015) [38]	Iran	31; 29	12 weeks; NA	Fasting blood glucose > 7 mmol/L	Int: 54.1 ± 8 Ctrl: 51.3 ± 7.7	NA	Iranian	Vit D-fortified doogh ** (with 170 mg calcium)	41.6 ± 15.9	77.1 ± 19.7	Unfortified doogh ** (170 mg Calcium)	41.1 ± 20.5	36.2 ± 23.6
Moreira-Lucas et al. (2016) [63]	Canada	35; 36	24 weeks; NA	With serum 25(OH)D ≤ 65 nmol/L, impaired fasting glucose and elevated glycated haemoglobin	Int: 49.1 ± 13.9 Ctrl: 45.6 ± 14.3	Int: 30.1 ± 3.9 Ctrl: 31.7 ± 4.9	White: Int *n* = 12 Ctrl *n* = 19	Vit D_3_-fortified low-fat cheddar cheese; 100	48.1 ± 14.3	98.7 ± 37.1	Unfortified low-fat cheddar cheese	47.6 ± 14.3	45.5 ± 14.3
Keane et al. (1992) [52]	Ireland	39; 39	3 months; NA	Elderly long-stay patients	Int: 85 Ctrl: 84	NA	NA	Vit D-fortified milk; 5	6.0 ± 6.4 ^3^	37.0 ± 23.1 ^3^	Unfortified milk	7.8 ± 9.2 ^3^	17.5 ± 11.2 ^3^
Nikooyeh et al. (2023) [49]	Iran	27; 27	8–12 weeks; Autumn and winter	T2DM patient	Int 45.0 ± 5.6 Ctrl: 44.1 ± 6.6	Int: 28.4 ± 4.0 Ctrl: 28.2 ± 3.7	NA	Vit D-fortified yoghurt drink; 25	39.7 ± 13.6	70.6 ± 19.2	Unfortified yoghurt drink	36.9 ± 18.9	32.9 ± 25.2
Sharifan et al. (2023) [50]	Iran	Int: Milk: 73 Yoghurt: 69; Ctrl: Milk: 73 Yoghurt: 74	10 weeks; winter	Abdominally obese	Int: Milk 40.42 ± 8.03 Yoghurt: 43.47 ± 7.21; Ctrl: Milk 40.26 ± 8.27 Yoghurt: 43.19 ± 7.21	Int: Milk 22.95 ± 2.96 Yoghurt: 23.8 ± 3.65 Ctrl: Milk 23.11 ± 3.16 Yoghurt: 23.27 ± 3.27	NA	Vit D_3_-fortified milk and Vit D_3_-fortified yoghurt; Milk and yoghurt: 37.5	Milk: 34.675 ± 13.05 Yoghurt: 35.5 ± 12.625	Milk: 47.825 ± 14.5 Yoghurt: 52.775 ± 14.225	Plain milk and yoghurt	Milk: 35.65 ± 12.6 Yoghurt: 38.55 ± 14.35	Milk: 35.4 ± 14.225 Yoghurt: 36.175 ± 9.925

* Also fortified with EPA, DHA, vitamins A, B1, B2, B3, pantothenic acid, B6, biotin, folic acid, B12, C, and E; ** a traditional Iranian fermented dairy product (yoghurt drink); ^1^ estimated from geometric mean (95% CI); ^2^ estimated from median and range; ^3^ converted 95% CI to SD; ^4^ estimated SD according to Cochrane handbook for missing SD; ^5^ SD estimated from SE or SEM. Int—intervention; Ctrl—control; BMI—body mass index; Conc—concentration; DC—vitamin D-fortified cheddar cheese; DLF—fortified low-fat cheese; Vit—vitamin; T2DM—type 2 diabetes mellitus; SD—standard deviation; SE—standard error; SEM—standard error of mean; MetS—metabolic syndrome; maxC—maximum concentration; PSST—premenstrual symptoms screening tool; 25(OH)D—25-hydroxyvitamin D; 25(OH)D_3_—25-hydroxyvitamin D_3_; GDM—gestational diabetes mellitus; NA—not available.

## Data Availability

The raw data supporting the conclusions of this article will be made available by the authors on request.

## Supplementary Materials

The following supporting information can be downloaded at https://www.mdpi.com/article/10.3390/nu17233757/s1, S1: Preferred Reporting Items for Systematic Reviews and Meta-Analyses (PRISMA) Checklist; S2: Search strategies; Figure S1: Risk of bias assessment; Table S1: Fortification methods and vitamin D concentrations of vitamin D-fortified dairy products in the included 35 RCTs; Table S2: Results of subgroup analysis of RCTs regarding the effect of vitamin D-fortified milk/milk powder on serum 25(OH)D concentration; Table S3: Results of subgroup analysis of RCTs regarding the effect of vitamin D-fortified yoghurt/yoghurt drinks on serum 25(OH)D concentration; Table S4: Results of subgroup analysis of RCTs regarding the effect of vitamin D-fortified cheese on serum 25(OH)D concentration; Table S5: Results of subgroup analysis of the 35 included RCTs regarding the effect of vitamin D-fortified dairy products on serum 25(OH)D concentration; Figure S2: Leave-one-out analysis of RCTs regarding the effect of vitamin D-fortified milk/milk powder on serum 25(OH)D concentration. MD—mean difference; Figure S3: Leave-one-out analysis of RCTs regarding the effect of vitamin D-fortified yoghurt/yoghurt drinks on serum 25(OH)D concentration. MD—mean difference; Figure S4: Leave-one-out analysis of RCTs regarding the effect of vitamin D-fortified cheese on serum 25(OH)D concentration. MD—mean difference; Figure S5: Relationship between the mean difference of serum 25(OH)D and serum 25(OH)D of participants at baseline (*p* = 0.074). Each bubble represents a data point, and its size is proportional to study weights (inverse-variance); Figure S6: Relationship between the mean difference of serum 25(OH)D and body mass index (adult) of participants at baseline (*p* < 0.001). Each bubble represents a data point, and its size is proportional to study weights (inverse-variance); Figure S7. Funnel plot for 35 included RCTs with 46 data points; Figure S8. Funnel plots by the types of dairy products and vitamin D; Figure S9. Funnel plots by the types of dairy products; Figure S10. Funnel plots by the number of participants. (a) RCTs with participants ≥ 100. (b) RCTs with participants < 100; Table S6. Results of Egger’s test, Begg’s test, and trim-and-fill analysis regarding publication bias; Table S7. Certainty of evidence assessed by the Grades of Recommendation, Assessment, Development and Evaluation (GRADE) approach.
