# Supplementary material for: Is Vitamin D Fortification of Dairy Products Effective for Improving Vitamin D Status? A Systematic Review and Meta-Analysis of Randomised Controlled Trials
Source: Nutrients. 2025 Nov 29;17(23):3757. doi: 10.3390/nu17233757 (PMC12694000; doi:10.3390/nu17233757)
Supplement: Supplementary file 1 [file nutrients-17-03757-s001.zip › nutrients-3974170-supplementary.pdf]

Supplementary Materials

S1. Preferred Reporting Items for Systematic Reviews and Meta-Analyses (PRISMA) Checklist

Section and Topic	Item #	Checklist item	Location where item is reported
**TITLE**			
Title	1	Identify the report as a systematic review.	Page 1
**ABSTRACT**			
Abstract	2	See the PRISMA 2020 for Abstracts checklist.	Page 1
**INTRODUCTION**			
Rationale	3	Describe the rationale for the review in the context of existing knowledge.	Page 2
Objectives	4	Provide an explicit statement of the objective(s) or question(s) the review addresses.	Page 2
**METHODS**			
Eligibility criteria	5	Specify the inclusion and exclusion criteria for the review and how studies were grouped for the syntheses.	Page 3
Information sources	6	Specify all databases, registers, websites, organisations, reference lists and other sources searched or consulted to identify studies. Specify the date when each source was last searched or consulted.	Page 3
Search strategy	7	Present the full search strategies for all databases, registers and websites, including any filters and limits used.	Page 3
Selection process	8	Specify the methods used to decide whether a study met the inclusion criteria of the review, including how many reviewers screened each record and each report retrieved, whether they worked independently, and if applicable, details of automation tools used in the process.	Page 3
Data collection process	9	Specify the methods used to collect data from reports, including how many reviewers collected data from each report, whether they worked independently, any processes for obtaining or confirming data from study investigators, and if applicable, details of automation tools used in the process.	Page 4
Data items	10a	List and define all outcomes for which data were sought. Specify whether all results that were compatible with each outcome domain in each study were sought (e.g. for all measures, time points, analyses), and if not, the methods used to decide which results to collect.	Page 4
	10b	List and define all other variables for which data were sought (e.g. participant and intervention characteristics, funding sources). Describe any assumptions made about any missing or unclear information.	Page 4
Study risk of bias assessment	11	Specify the methods used to assess risk of bias in the included studies, including details of the tool(s) used, how many reviewers assessed each study and whether they worked independently, and if applicable, details of automation tools used in the process.	Page 4
Effect measures	12	Specify for each outcome the effect measure(s) (e.g. risk ratio, mean difference) used in the synthesis or presentation of results.	Page 4-5
Synthesis	13a	Describe the processes used to decide which studies were eligible for each synthesis (e.g. tabulating the study intervention	Page 4-5

Section and Topic	Item #	Checklist item	Location where item is reported
methods		characteristics and comparing against the planned groups for each synthesis (item #5)).	
	13b	Describe any methods required to prepare the data for presentation or synthesis, such as handling of missing summary statistics, or data conversions.	Page 4-5
	13c	Describe any methods used to tabulate or visually display results of individual studies and syntheses.	Page 4-5
	13d	Describe any methods used to synthesize results and provide a rationale for the choice(s). If meta-analysis was performed, describe the model(s), method(s) to identify the presence and extent of statistical heterogeneity, and software package(s) used.	Page 4-5
	13e	Describe any methods used to explore possible causes of heterogeneity among study results (e.g. subgroup analysis, meta-regression).	Page 4-5
	13f	Describe any sensitivity analyses conducted to assess robustness of the synthesized results.	Page 4-5
Reporting bias assessment	14	Describe any methods used to assess risk of bias due to missing results in a synthesis (arising from reporting biases).	Page 4-5
Certainty assessment	15	Describe any methods used to assess certainty (or confidence) in the body of evidence for an outcome.	Page 5
**RESULTS**			
Study selection	16a	Describe the results of the search and selection process, from the number of records identified in the search to the number of studies included in the review, ideally using a flow diagram.	Page 5
	16b	Cite studies that might appear to meet the inclusion criteria, but which were excluded, and explain why they were excluded.	Page 5-6
Study characteristics	17	Cite each included study and present its characteristics.	Page 7-14
Risk of bias in studies	18	Present assessments of risk of bias for each included study.	Page 7-8
Results of individual studies	19	For all outcomes, present, for each study: (a) summary statistics for each group (where appropriate) and (b) an effect estimate and its precision (e.g. confidence/credible interval), ideally using structured tables or plots.	Page 8-14
Results of syntheses	20a	For each synthesis, briefly summarise the characteristics and risk of bias among contributing studies.	Page 6-17
	20b	Present results of all statistical syntheses conducted. If meta-analysis was done, present for each the summary estimate and its precision (e.g. confidence/credible interval) and measures of statistical heterogeneity. If comparing groups, describe the direction of the effect.	Page 15-17
	20c	Present results of all investigations of possible causes of heterogeneity among study results.	supplementary materials
	20d	Present results of all sensitivity analyses conducted to assess the robustness of the synthesized results.	Page 17-18, supplementary materials
Reporting biases	21	Present assessments of risk of bias due to missing results (arising from reporting biases) for each synthesis assessed.	Page 17-18, supplementary

Section and Topic	Item #	Checklist item	Location where item is reported
			materials
Certainty of evidence	22	Present assessments of certainty (or confidence) in the body of evidence for each outcome assessed.	Page 18, supplementary materials
**DISCUSSION**			
Discussion	23a	Provide a general interpretation of the results in the context of other evidence.	Page 18-21
	23b	Discuss any limitations of the evidence included in the review.	Page 21
	23c	Discuss any limitations of the review processes used.	Page 21
	23d	Discuss implications of the results for practice, policy, and future research.	Page 21-22
**OTHER INFORMATION**			
Registration and protocol	24a	Provide registration information for the review, including register name and registration number, or state that the review was not registered.	Page 3
	24b	Indicate where the review protocol can be accessed, or state that a protocol was not prepared.	Page 3
	24c	Describe and explain any amendments to information provided at registration or in the protocol.	Page 3
Support	25	Describe sources of financial or non-financial support for the review, and the role of the funders or sponsors in the review.	Page 22-23
Competing interests	26	Declare any competing interests of review authors.	Page 22-23
Availability of data, code and other materials	27	Report which of the following are publicly available and where they can be found: template data collection forms; data extracted from included studies; data used for all analyses; analytic code; any other materials used in the review.	Page 22-23

From: Page MJ, McKenzie JE, Bossuyt PM, Boutron I, Hoffmann TC, Mulrow CD, et al. The PRISMA 2020 statement: an updated guideline for reporting systematic reviews. BMJ 2021;372:n71. doi: 10.1136/bmj.n71. This work is licensed under CC BY 4.0. To view a copy of this license, visit https://creativecommons.org/licenses/by/4.0/

S2. Search strategies

1. ***Keywords for PubMed***

1	"vitamin d"[MeSH Terms] OR "vitamin d"[All Fields] OR "ergocalciferols"[MeSH Terms] OR "ergocalciferols"[All Fields] OR "25 oh d"[All Fields] OR "25-Hydroxyvitamin D"[All Fields] OR ("1 25 dihydroxyvitamin d"[Supplementary Concept] OR "1 25 dihydroxyvitamin d"[All Fields] OR "1 25 dihydroxyvitamin d"[All Fields])
2	"fortification"[All Fields] OR "fortifications"[All Fields] OR "food, fortified"[MeSH Terms]
3	"Dairy Products"[MeSH Terms] OR ("yoghurts"[All Fields] OR "yogurt"[MeSH Terms] OR "yogurt"[All Fields] OR "yoghurt"[All Fields] OR "yogurts"[All Fields]) OR ("milk, human"[MeSH Terms] OR ("milk"[All Fields] AND "human"[All Fields]) OR "human milk"[All Fields] OR "milk"[All Fields] OR "milk"[MeSH Terms]) OR ("cheese"[MeSH Terms] OR "cheese"[All Fields] OR "cheeses"[All Fields] OR "cheese s"[All Fields])
4	("randomized controlled trial"[Publication Type] OR "randomized controlled trials as topic"[MeSH Terms] OR "randomized controlled trials"[All Fields] OR "randomised controlled trials"[All Fields]) AND (("human s"[All Fields] OR "humans"[MeSH Terms] OR "humans"[All Fields] OR "human"[All Fields]) AND "stud*"[All Fields])
5	(#1 AND #2 AND #3) AND #4

2. ***Keywords for Embase***

**1**	exp vitamin D/
**2**	("Vitamin D" or "vitamin d2" or "vitamin d3" or Cholecalciferol or Ergocalciferols or "25-Hydroxyvitamin D2" or "25-Hydroxyvitamin D" or "1,25-Dihydroxyvitamin D").mp. [mp=title, abstract, heading word, drug trade name, original title, device manufacturer, drug manufacturer, device trade name, keyword heading word, floating subheading word, candidate term word]
**3**	exp fortified food/
**4**	fortif*.mp. [mp=title, abstract, heading word, drug trade name, original title, device manufacturer, drug manufacturer, device trade name, keyword heading word, floating subheading word, candidate term word]
**5**	exp dairy product/
**6**	exp "randomized controlled trial (topic)"/
**7**	("randomi* controlled trial*" or "clinical trial*" or "clinical controlled trial*" or "RCT*" or intervention* or placebo*).mp. [mp=title, abstract, heading word, drug trade name, original title, device manufacturer, drug manufacturer, device trade name, keyword heading word, floating subheading word, candidate term word]
**8**	1 OR 2
**9**	(3 OR 4) AND 5
**10**	(6 OR 7) AND 8
**11**	(9 AND 10) AND 11
**12**	Limit 11 to human

3. ***Keywords for Web of Science***

**1**	ALL=(“Vitamin D” OR “vitamin d2” OR “vitamin d3” OR Cholecalciferol OR Ergocalciferols OR “25-Hydroxyvitamin D2” OR “25-Hydroxyvitamin D” OR “1,25-Dihydroxyvitamin D”)
**2**	ALL=(fortif* OR fortified food)
**3**	ALL=(dairy OR dairy products OR milk OR cheese* OR cream* OR yog*urt OR milk-derived)
**4**	ALL=("randomi* controlled trial*" OR "clinical trial*" OR "clinical controlled trial*" OR “RCT*” OR intervention* or placebo*)
**5**	ALL=(human)
**6**	#1 AND (#2 AND #3)
**7**	#4 AND #5
**8**	#6 AND #7

S3. Risk of Bias Assessment

Study	D1	D2	D3	D4	D5	Overall
Bonjour et al. (2013)	+	!	+	+	!	!
Brett et al. (2018)	+	+	+	+	+	+
Du et al. (2004)	!	-	!	+	+	-
Fisk et al. (2012)	!	+	+	+	!	!
Ganmaa et al. (2017)	+	+	+	+	+	+
Green et al. (2010)	+	+	+	+	!	!
Hajimohammadi et al. (2017)	!	!	+	+	+	!
Hower et al. (2013)	+	+	+	+	!	!
Jafari et al. (2016)	+	+	+	+	+	+
Johnson et al. (2005)	-	!	+	+	!	-
Keane et al. (1992)	!	+	+	+	!	!
Khadgawat et al. (2013)	+	+	+	+	!	!
Kruger et al. (2016)	+	!	+	+	!	!
Kruger et al. (2018)	+	!	+	+	!	!
Li and Xing (2016)	+	+	+	+	!	!
Lovell et al. (2018)	+	+	+	+	+	+
Manios et al. (2017)	+	+	+	+	+	+
Marwaha et al. (2021)	+	+	+	+	!	!
McKenna et al. (1995)	+	!	+	+	!	!
Mohammadi-Sartang et al. (2018)	+	+	+	+	+	+
Moreira-Lucas et al. (2016)	+	+	+	+	+	+
Neyestani et al. (2012)	+	!	!	+	!	!
Neyestani et al. (2014)	+	!	+	+	!	!
Nikooyeh et al. (2011)	+	!	+	+	!	!
Nikooyeh et al. (2023)	+	+	+	+	+	+
Petrova et al. (2019)	+	+	+	+	!	!
Romeo et al. (2011)	+	!	+	+	!	!
Shab-Bidar et al. (2011)	+	!	+	+	!	!
Shab-Bidar et al. (2015)	+	!	+	+	+	!
Sharifan et al. (2021)	+	+	+	+	!	!
Sharifan et al. (2023)	+	+	+	+	+	+
Sharifan et al. (2024)	+	!	+	+	+	!
Taghizadeh et al. (2021)	+	+	+	+	+	+
Toxqui et al. (2014)	+	+	+	+	!	!
Wagner et al. (2008)	+	+	+	+	!	!

+ Low risk
! Some concerns
- High risk

D1 Randomisation process
D2 Deviations from the intended interventions
D3 Missing outcome data
D4 Measurement of the outcome
D5 Selection of the reported result

Figure S1. Risk of bias assessment.

S4. Characteristics of Studies

Table S1. Fortification methods and vitamin D concentrations of vitamin D-fortified dairy products in the included 35 RCTs.

Study	Type of vitamin D	Type of dairy products	Vitamin D concentrations	Fortification method
McKenna et al.[1]	Vitamin D_3	Milk	1.2 μg/100 mL	NA
Du et al.[2]	Vitamin D_3	Milk	1.5 μg/100 mL	NA
Johnson et al.[3]	Vitamin D_3	Cheese	17.6 μg/100 g	NA
Green et al.[4]	Vitamin D_3	Milk powder	6.7 μg/100 g	Provided by dairy product manufacturer
Nikooyeh et al.[5]	Vitamin D_3	Yoghurt drink	5 μg/100 mL	NA
Romeo et al.[6]	Vitamin D	Milk	0.75 μg/100 mL	NA
Shab-Bidar et al.[7]	Vitamin D_3	Doogh*	5 μg/100 mL	NA
Fisk et al.[8]	Vitamin D_2 and D_3	Malted milk drink	D_2: 19.2 and 30 μg/100 g<br>D_3: 20.8 and 40 μg/100 g	Provided by dairy product manufacturer
Neyestani et al.[9]	Vitamin D_3	Doogh*	5 μg/100 mL	NA
Bonjour et al.[10]	Vitamin D_3	Yoghurt	8 μg/100 g	Direct mixing with vitamin D
Hower et al.[11]	Vitamin D	Growing up milk	2.85 μg/100 mL	Provided by dairy product manufacturer
Khadgawat et al.[12]	Vitamin D_3	Milk	7.5 μg/100 mL<br>12.5 μg/100 mL	Direct mixing with vitamin D
Neyestani et al.[13]	Vitamin D	Milk	1.25 μg/100 mL	NA
Toxqui et al.[14]	Vitamin D_3	Milk	1 μg/100 mL	Provided by dairy product manufacturer
Jafari et al.[15]	Vitamin D_3	Yoghurt	50 μg/100g	Provided by dairy product manufacturer
Kruger et al.[16]	Vitamin D	Milk	15 μg/serving	Provided by dairy product manufacturer
Li and Xing[17]	Vitamin D_3	Yoghurt	12.5 μg/100g	Provided by dairy product manufacturer
Hajimohammadi et al.[18]	Vitamin D	Yoghurt drink	5 μg/100 mL	NA
Manios et al.[19]	Vitamin D_3	Cheese	9.5 μg/100 g	NA
Brett et al.[20]	Vitamin D_3	Cheese, yoghurt	Cheese: 22.7 μg/100 g<br>Yoghurt: 4.03 μg/100 mL	Provided by dairy product manufacturer
Kruger et al.[21]	Vitamin D	Milk powder	15 μg/serving	Provided by dairy product manufacturer
Lovell et al.[22]	Vitamin D_3	Growing-up milk	1.2 μg/100 mL	NA
Mohammadi-Sartang et al.[23]	Vitamin D_3	Yoghurt	5 μg/100 g	Provided by dairy product manufacturer
Petrova et al.[24]	Vitamin D	Milk	0.75 μg/100 mL	Provided by dairy product manufacturer
Marwaha et al.[25]	Vitamin D_2	Milk	3 μg/100 mL	Provided by dairy product manufacturer
Sharifan et al.[26]	Vitamin D_3	Milk, yoghurt	Milk: 18.8 μg/100 mL<br>Yoghurt: 25 μg/100 g	Nanoencapsulation of vitamin D
Taghizadeh et al.[27]	Vitamin D	Yoghurt	325 μg/100 g	Nanoencapsulation of vitamin D

Table S1. Continued.

Sharifan et al.[28]	Vitamin D_3	Milk	Milk: 18.8 µg/100 mL<br>Yoghurt: 25 µg/100 g	Nanoencapsulation of vitamin D
Wagner et al.[29]	Vitamin D_3	Cheese	Regular: 298 µg/100 g<br>Low-fat: 242 µg/100 g	Provided by dairy product manufacturer
Keane et al.[30]	Vitamin D_3	Milk	1 µg/100 mL	NA
Shab-Bidar et al.[31]	Vitamin D_3	Doogh*	5 µg/100 mL	NA
Moreira-Lucas et al.[32]	Vitamin D_3	Cheese	333 µg/100 g	Provided by dairy product manufacturer
Ganmaa et al.[33]	Vitamin D	Milk	1.06 µg/100 mL	Provided by dairy product manufacturer
Nikooyeh et al.[34]	Vitamin D	Yoghurt drink	5 µg/100 mL	NA
Sharifan et al.[35]	Vitamin D_3	Milk, yoghurt	Milk: 18.8 µg/100 mL<br>Yoghurt: 25 µg/100 mL	Nanoencapsulation of vitamin D

*A traditional Iranian fermented dairy product (yoghurt drink); NA,—not available. Note: Vitamin D without a subscript (i.e. D_2 or D_3) indicates the specific type is not reported by researchers.

S5. Subgroup Analysis

Table S2. Results of subgroup analysis of RCTs regarding the effect of vitamin D-fortified milk/milk powder on serum 25(OH)D concentration.

Subgroup	Study (n)	MD (nmol/L)	95% CI (nmol/L)		P-value	I^2 (%)
			Lower	Upper		
Vitamin D dose (μg/day)						
<25	17	16.5	12.7	20.3	<0.001	88.6
≥25	9	15.7	8.7	22.4	<0.001	99.2
Serum 25(OH)D at baseline (nmol/L)						
Deficient (<25)	4	21.4	11.6	31.2	<0.001	94.4
Insufficient (≥25 & 50)	12	17.7	11.9	23.5	<0.001	96.8
Sufficient (>50)	10	11.0	9.0	12.9	<0.001	69.6
Age at baseline (years)						
Adult (≥18)	16	12.8	10.2	15.4	<0.001	90.3
Child and teenager (<18)	10	19.9	13.0	26.8	<0.001	97.3
Risk of bias level						
High risk	1	26.8	22.8	30.8	<0.001	NA
Low risk	3	15.9	1.46	30.2	0.031	98.4
Some concerns	22	15.6	12.0	19.2	<0.001	96.2
Body mass index at baseline (adult)						
Normal	11	10.6	7.9	13.2	<0.001	88.8
NA	5	17.1	14.5	19.7	<0.001	0
Continents						
Asia	14	17.4	12.0	22.9	<0.001	98.9
Europe	10	13.4	11.4	15.5	<0.001	0
Oceania	2	11.9	5.1	18.7	0.001	0
Production/Fortification methods						
Direct mixing with vitamin D	2	34.6	20.1	47.3	<0.001	98.0
NA	6	16.2	9.9	22.5	<0.001	81.3
Nanoencapsulation of vitamin D	5	13.2	8.3	18.0	<0.001	88.4
Provided by dairy product manufacturer	13	13.9	10.1	17.7	<0.001	95.2

MD—mean difference; CI—confidence interval; NA—not available.

Table S3. Results of subgroup analysis of RCTs regarding the effect of vitamin D-fortified yoghurt/yoghurt drinks on serum 25(OH)D concentration.

Subgroup	Study (n)	MD (nmol/L)	95% CI (nmol/L)		P-value	I^2 (%)
			Lower	Upper		
Vitamin D dose (μg/day)						
<25	2	12.2	-3.6	27.9	0.13	91.5
≥25	13	28.8	22.8	34.7	<0.001	95.8
Serum 25(OH)D at baseline (nmol/L)						
Deficient (<25)	1	20.1	14.2	26.0	<0.001	NA
Insufficient (≥25 & 50)	11	28.5	21.4	35.6	<0.001	95.3
Sufficient (>50)	3	22.1	4.7	39.5	0.01	97.2
Age at baseline (years)						
Adult (≥18)	14	28.1	22.5	33.7	<0.001	95.6
Child and teenager (<18)	1	4.0	-3.0	11.0	0.27	NA
Risk of bias level						
Low risk	7	20.5	11.9	29.1	<0.001	97.7
Some concerns	8	32.1	25.3	38.9	<0.001	86.0
Body mass index at baseline (adult)						
Normal	6	21.1	13.3	28.9	<0.001	95.7
NA	1	40.2	31.3	49.1	<0.001	NA
Overweight	7	32.3	16.7	38.0	<0.001	79.6
Continents						
Asia	13	28.8	22.8	34.7	<0.001	95.5
Europe	1	20.1	14.2	26.0	<0.001	NA
North America	1	4.0	-3.0	11.0	0.27	NA
Production/Fortification methods						
Direct mixing with vitamin D	1	20.1	14.2	26.0	<0.001	NA
NA	6	39.0	34.9	43.2	<0.001	0
Nanoencapsulation of vitamin D	4	16.1	10.1	22.1	<0.001	91.6
Provided by dairy product manufacturer	4	24.8	11.6	38.0	<0.001	97.2

MD—mean difference; CI—confidence intervals; NA—not available.

Table S4. Results of subgroup analysis of RCTs regarding the effect of vitamin D-fortified cheese on serum 25(OH)D concentration.

Subgroup	Study (n)	MD (nmol/L)	95% CI (nmol/L)		P-value	I^2 (%)
			Lower	Upper		
Vitamin D dose (μg/day)						
<25	2	-0.2	-19.5	19.2	0.99	99.5
≥25	3	30.0	6.4	53.6	0.01	82.6
Serum 25(OH)D at baseline (nmol/L)						
Insufficient (≥25 & 50)	2	30.6	-11.5	72.7	0.15	97.0
Sufficient (>50)	3	6.4	-13.3	26.2	0.52	84.9
Age at baseline (years)						
Adult (≥18)	5	16.8	-3.6	37.2	0.11	99.3
Risk of bias level						
High risk	1	-10.0	-10.8	-9.2	<0.001	NA
Low risk	2	30.6	-11.5	72.7	0.15	97.0
Some concerns	2	17.7	4.7	30.8	0.01	0.00
Body mass index at baseline (adult)						
Normal	1	16.2	-3.6	36.0	0.11	NA
NA	1	-10.0	-10.8	-9.2	<0.001	NA
Obese	1	52.7	38.7	67.0	<0.001	NA
Overweight	2	10.1	6.5	13.7	<0.001	4.5
Continents						
Europe	1	9.7	7.2	12.2	<0.001	NA
North America	4	18.9	-7.3	45.1	0.16	94.7
Production/Fortification methods						
NA	2	-0.2	-19.5	19.2	0.99	99.5
Provided by dairy product manufacturer	3	30.0	6.4	53.9	0.01	82.6

MD—mean difference; CI—confidence interval; NA—not available.

Table S5. Results of subgroup analysis of the 35 included RCTs regarding the effect of vitamin D-fortified dairy products on serum 25(OH)D concentration.

Subgroup	Study (n)	MD (nmol/L)	95% CI (nmol/L)		P-value	I^2 (%)
			Lower	Upper		
Vitamin D dose (µg/day)						
<25	21	14.1	9.9	18.3	<0.001	96.1
≥25	25	23.9	18.8	29.0	<0.001	98.8
Serum 25(OH)D at baseline (nmol/L)						
Deficient (<25)	5	21.2	13.5	28.9	<0.001	92.4
Insufficient (≥25 & 50)	25	23.1	18.1	28.2	<0.001	97.8
Sufficient (>50)	16	12.8	7.5	18.0	<0.001	98.7
Age at baseline (years)						
Adult (≥18)	35	19.7	15.5	23.9	<0.001	98.8
Child and teenager (<18)	11	18.5	11.6	25.3	<0.001	97.3
Risk of bias level						
High risk	2	8.3	-27.7	44.4	0.65	99.7
Low risk	12	20.6	12.9	28.4	<0.001	98.9
Some concerns	32	19.6	15.9	23.4	<0.001	96.9
Body mass index at baseline (adult)						
Normal	18	14.9	11.2	18.6	<0.001	97.1
NA	7	16.3	5.2	27.4	0.004	97.6
Obese	1	52.7	38.4	67.0	<0.001	NA
Overweight	9	28.5	21.2	35.9	<0.001	93.9
Continents						
Asia	27	22.6	18.2	27.1	<0.001	98.7
Europe	12	13.9	11.2	16.7	<0.001	51.7
North America	5	15.6	-5.3	36.6	0.144	96.9
Oceania	2	11.9	5.1	18.7	0.001	0
Vitamin D type						
Vitamin D (type not specified)	12	17.9	12.2	23.7	<0.001	98.2
Vitamin D_2	3	11.6	9.3	13.9	<0.001	0
Vitamin D_3	31	20.7	15.9	25.4	<0.001	98.5
Production/Fortification methods						
Direct mixing with vitamin D	3	29.9	17.6	42.2	<0.001	97.4
NA	14	22.4	13.9	30.8	<0.001	97.9
Nanoencapsulation of vitamin D	9	14.5	10.8	18.1	<0.001	93.3
Provided by dairy product manufacturer	20	18.1	13.2	23.1	<0.001	97.9

MD—mean difference; CI—confidence interval; NA—not available.

S5. Leave-one-out analysis

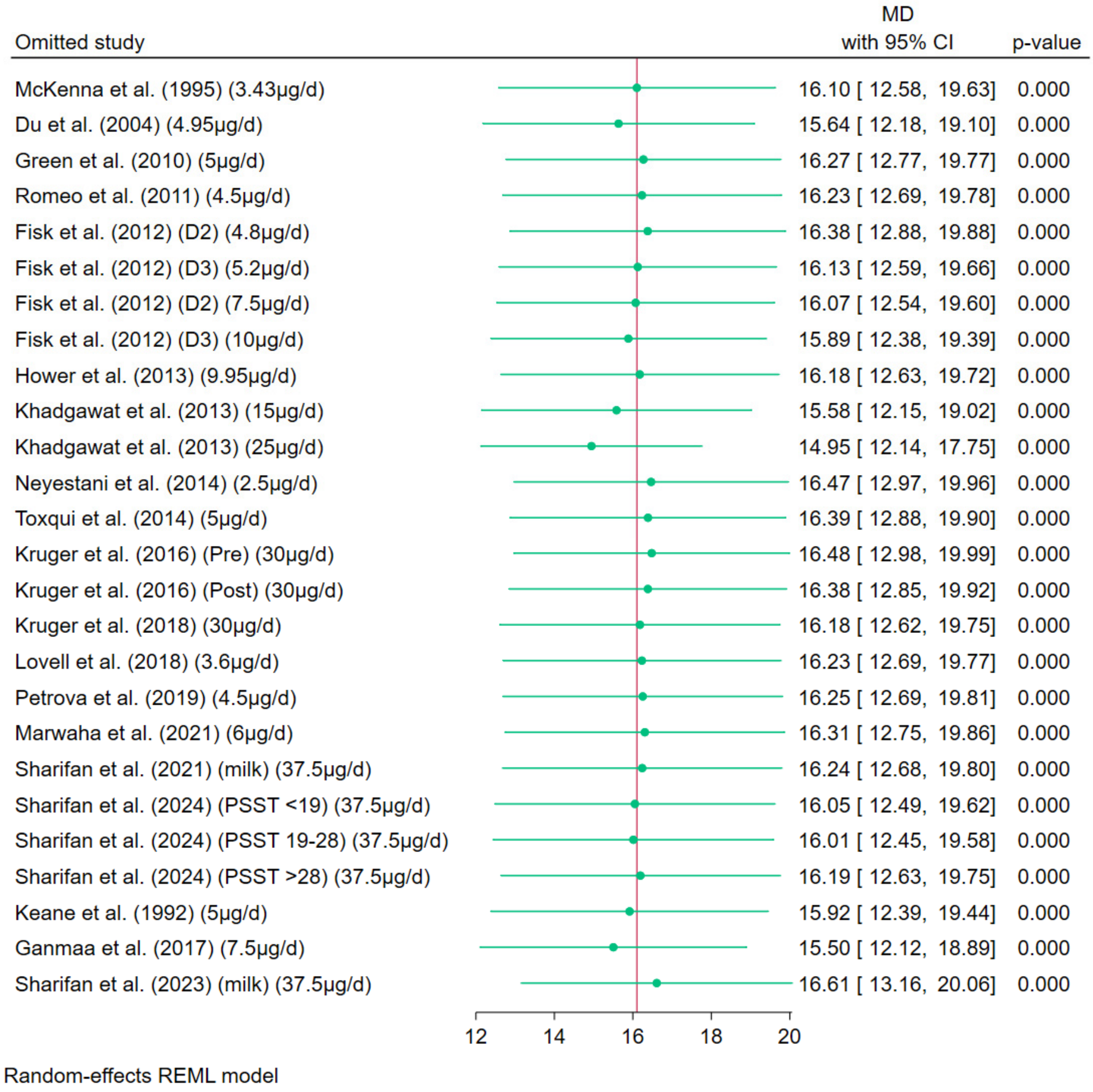

Figure S2. Leave-one-out analysis of RCTs regarding the effect of vitamin D-fortified milk/milk powder on serum 25(OH)D concentration. MD—mean difference.

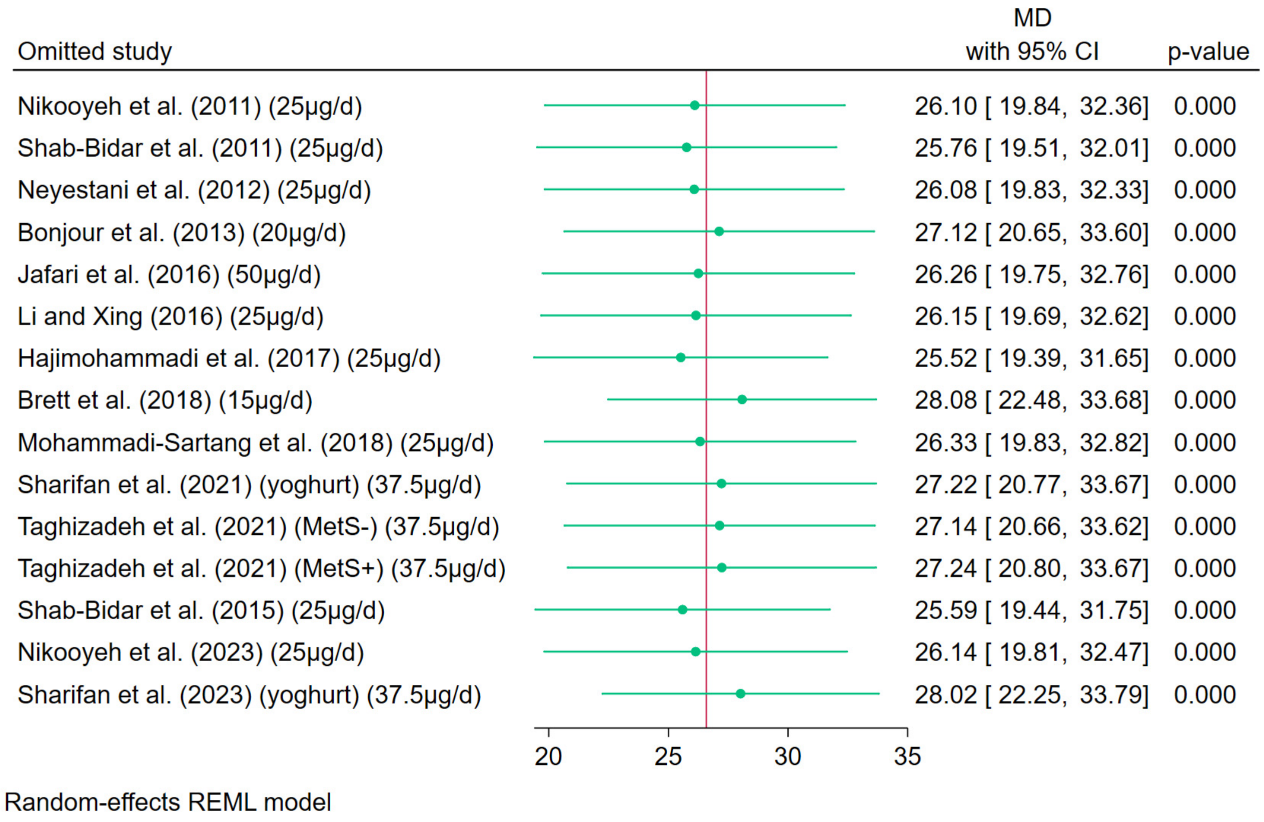

Figure S3. Leave-one-out analysis of RCTs regarding the effect of vitamin D-fortified yoghurt/yoghurt drinks on serum 25(OH)D concentration. MD—mean difference.

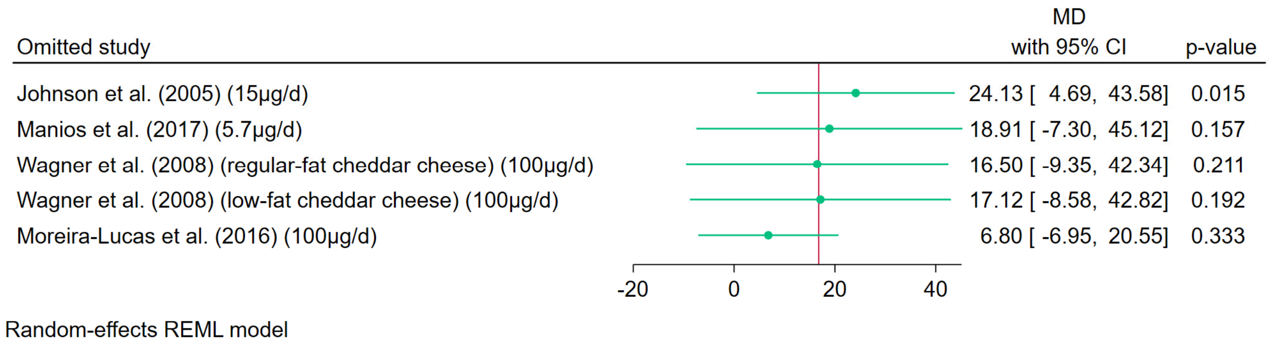

Figure S4. Leave-one-out analysis of RCTs regarding the effect of vitamin D-fortified cheese on serum 25(OH)D concentration. MD—mean difference.

S6. Meta-regression Analysis

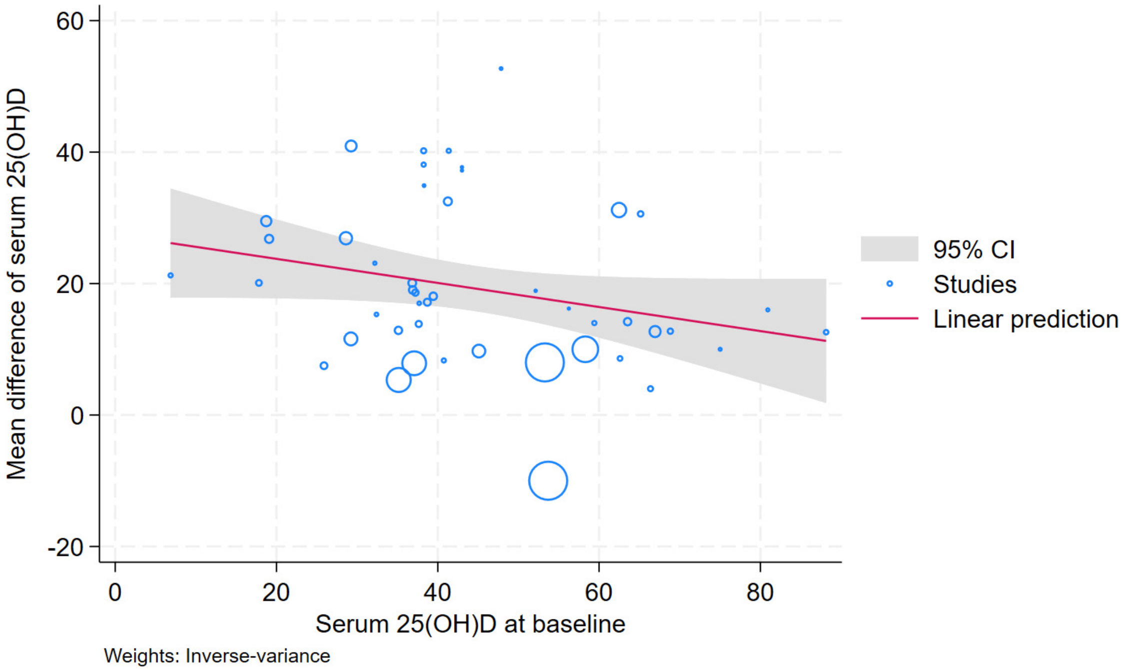

Figure S5. Relationship between the mean difference in serum 25(OH)D and serum 25(OH)D of participants at baseline (p = 0.074). Each bubble represents a data point, and its size is proportional to study weights (inverse-variance).

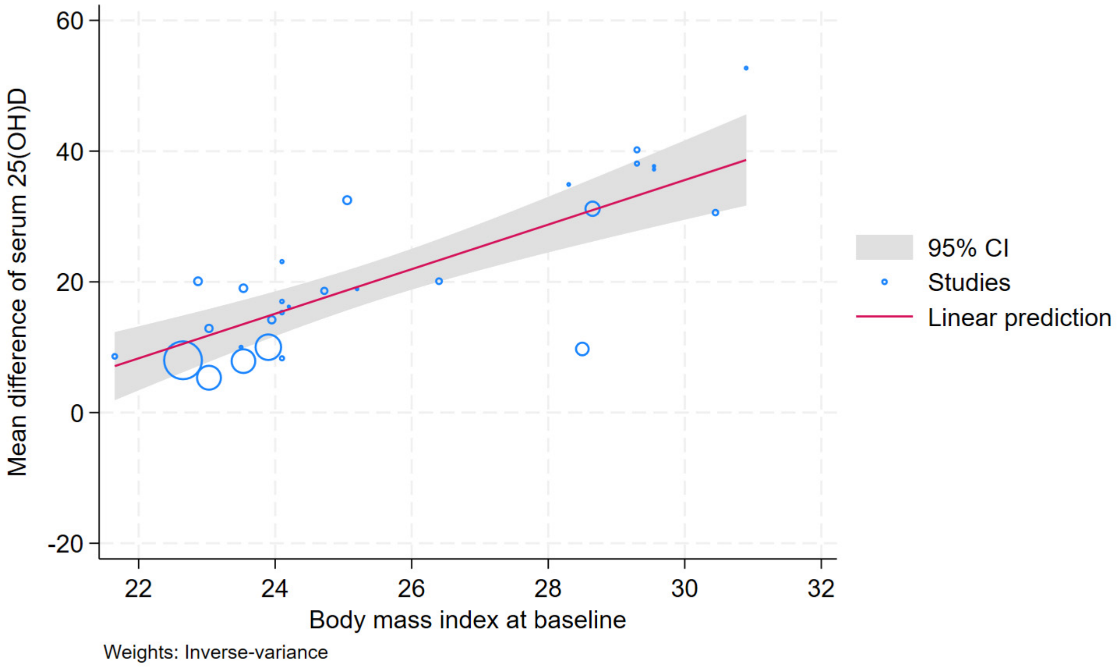

Figure S6. Relationship between the mean difference in serum 25(OH)D and body mass index (adult) of participants at baseline ($p < 0.001$). Each bubble represents a data point, and its size is proportional to study weights (inverse-variance).

S7. Publication Bias

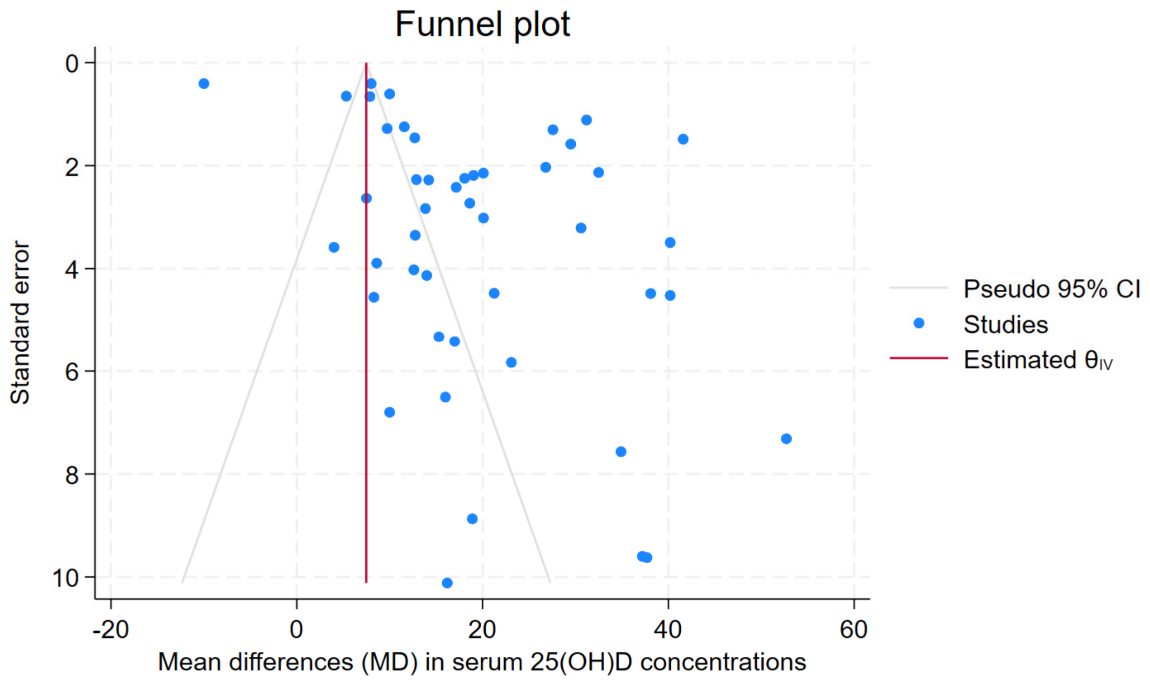

Figure S7. Funnel plot for 35 included RCTs with 46 data points.

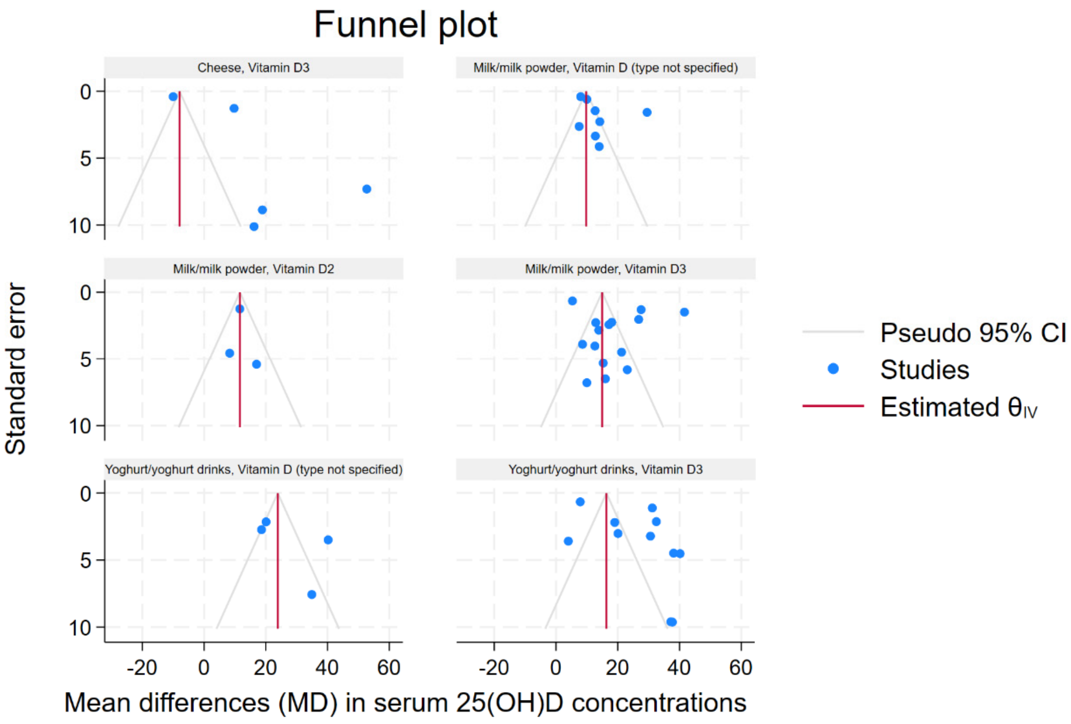

Figure S8. Funnel plots by the types of dairy products and vitamin D.

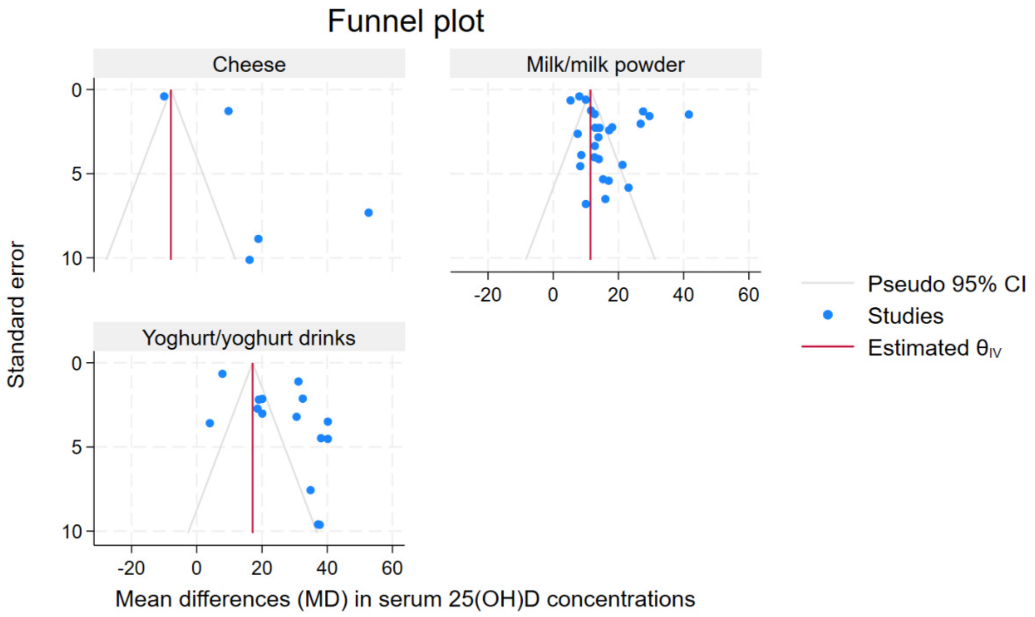

Figure S9. Funnel plots by the types of dairy products.

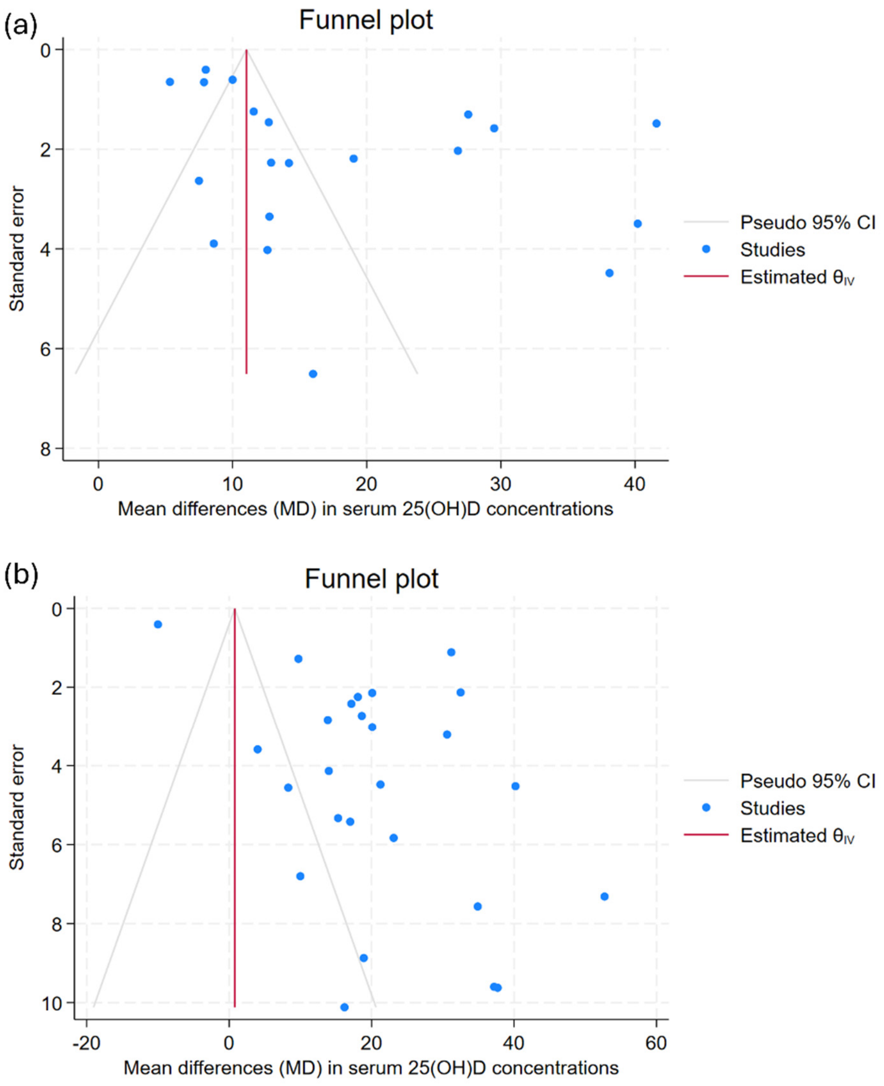

Figure S10. Funnel plots by the number of participants. (a) RCTs with participants ≥ 100. (b) RCTs with participants < 100.

Table S6. Results of Egger's test, Begg's test, and trim-and-fill analysis regarding publication bias.

**Intervention/Subgroups**	**Data point (n)**	**Egger's test (p-value)**	**Trim-and-fill**		
			**Imputed studies (n)**	**Adjusted MD (nmol/L)**	**Adjusted 95% CI (nmol/L)**
Vitamin D-fortified dairy products (overall)	46	0.016	0	19.34	15.80 – 22.89
Vitamin D-fortified milk/milk powder (overall)	26	0.753	NA	NA	NA
Vitamin D (type not specified)-fortified milk/milk powder	8	0.855	NA	NA	NA
Vitamin D_2-fortified milk/milk powder	3	0.800	NA	NA	NA
Vitamin D_3-fortified milk/milk powder	15	0.388	NA	NA	NA
Vitamin D-fortified yoghurt/yoghurt drinks (overall)	15	0.045	3	23.36	17.28 – 29.44
Vitamin D (type not specified)-fortified yoghurt/yoghurt drinks	4	0.332	NA	NA	NA
Vitamin D_3-fortified yoghurt/yoghurt drinks	11	0.104	NA	NA	NA
Vitamin D_3-fortified cheese (overall)	5	0.179	NA	NA	NA
RCTs with participants ≥ 100 (Sensitivity test)	20	0.342	NA	NA	NA
RCTs with participants < 100 (Sensitivity test)	26	0.037	0	20.51	15.51 – 25.52
RCTs without vitamin D-fortified yoghurt/yoghurt drink (Sensitivity test)	31	0.162	NA	NA	NA

MD—mean difference; CI—confidence intervals; NA—not available. Note: Egger's test ($p < 0.1$) indicates a potential publication bias.

S8. Certainty of Evidence

Table S7. Certainty of evidence assessed by the Grades of Recommendation, Assessment, Development and Evaluation (GRADE) approach.

Outcome	No. of Studies	Risk of Bias	Inconsistency	Indirectness	Imprecision	Other considerations	Certainty
Vitamin D-fortified milk/milk powder (Overall)	26	Serious	Very serious	Not serious	Not serious	Strong association, dose response gradient	⊕⊕⊕◯ Moderate
Vitamin D (type not specified)-fortified milk/milk powder	8	Serious	Very serious	Not serious	Very Serious	Strong association	⊕◯◯◯ Very low
Vitamin D_2-fortified milk/milk powder	3	Serious	Not serious	Not serious	Very serious	Dose response gradient	⊕⊕◯◯ Low
Vitamin D_3-fortified milk/milk powder	15	Serious	Very serious	Not serious	Not serious	Very strong association, dose response gradient	⊕⊕⊕⊕ High
Vitamin D-fortified yoghurt/yoghurt drinks (Overall)	15	Serious	Very serious	Not serious	Serious	Publication bias strongly suspected, very strong association, dose response gradient	⊕⊕◯◯ Low
Vitamin D (type not specified)-fortified yoghurt/yoghurt drinks	4	Serious	Very serious	Not serious	Very Serious	Very strong association	⊕◯◯◯ Very low
Vitamin D_3-fortified yoghurt/yoghurt drinks	11	Serious	Very serious	Not serious	Very Serious	Very strong association, dose response gradient	⊕⊕◯◯ Low
Vitamin D_3-fortified cheese (overall)	5	Serious	Very serious	Not serious	Extremely Serious	Strong association, dose response gradient	⊕◯◯◯ Very low

References

1. McKenna, M.J.; Freaney, R.; Byrne, P.; McBrinn, Y.; Murray, B.; Kelly, M.; Donne, B.; O'Brien, M. Safety and efficacy of increasing wintertime vitamin D and calcium intake by milk fortification. *Qjm* **1995**, *88*, 895–898.

2. Du, X.; Zhu, K.; Trube, A.; Zhang, Q.; Ma, G.; Hu, X.; Fraser, D.R.; Greenfield, H. School-milk intervention trial enhances growth and bone mineral accretion in Chinese girls aged 10-12 years in Beijing. *Br J Nutr* **2004**, *92*, 159–168, doi:10.1079/bjn20041118.

3. Johnson, J.L.; Mistry, V.V.; Vukovich, M.D.; Hogie-Lorenzen, T.; Hollis, B.W.; Specker, B.L. Bioavailability of vitamin D from fortified process cheese and effects on vitamin D status in the elderly. *J Dairy Sci* **2005**, *88*, 2295–2301, doi:10.3168/jds.S0022-0302(05)72907-6.

4. Green, T.J.; Skeaff, C.M.; Rockell, J.E. Milk fortified with the current adequate intake for vitamin D (5 microg) increases serum 25-hydroxyvitamin D compared to control milk but is not sufficient to prevent a seasonal decline in young women. *Asia Pac J Clin Nutr* **2010**, *19*, 195–199.

5. Nikooyeh, B.; Neyestani, T.R.; Farvid, M.; Alavi-Majd, H.; Houshiarrad, A.; Kalayi, A.; Shariatzadeh, N.; Gharavi, A.; Heravifard, S.; Tayebinejad, N.; et al. Daily consumption of vitamin D- or vitamin D + calcium-fortified yogurt drink improved glycemic control in patients with type 2 diabetes: a randomized clinical trial. *Am J Clin Nutr* **2011**, *93*, 764–771, doi:10.3945/ajcn.110.007336.

6. Romeo, J.; Wärnberg, J.; García-Mármol, E.; Rodríguez-Rodríguez, M.; Diaz, L.E.; Gomez-Martínez, S.; Cueto, B.; López-Huertas, E.; Cepero, M.; Boza, J.J.; et al. Daily consumption of milk enriched with fish oil, oleic acid, minerals and vitamins reduces cell adhesion molecules in healthy children. *Nutr Metab Cardiovasc Dis* **2011**, *21*, 113–120, doi:10.1016/j.numecd.2009.08.007.

7. Shab-Bidar, S.; Neyestani, T.R.; Djazayery, A.; Eshraghian, M.R.; Houshiarrad, A.; Gharavi, A.; Kalayi, A.; Shariatzadeh, N.; Zahedirad, M.; Khalaji, N.; et al. Regular consumption of vitamin D-fortified yogurt drink (Doogh) improved endothelial biomarkers in subjects with type 2 diabetes: a randomized double-blind clinical trial. *BMC Med* **2011**, *9*, 125, doi:10.1186/1741-7015-9-125.

8. Fisk, C.M.; Theobald, H.E.; Sanders, T.A. Fortified malted milk drinks containing low-dose ergocalciferol and cholecalciferol do not differ in their capacity to raise serum 25-hydroxyvitamin D concentrations in healthy men and women not exposed to UV-B. *J Nutr* **2012**, *142*, 1286–1290, doi:10.3945/jn.111.156166.

9. Neyestani, T.R.; Nikooyeh, B.; Alavi-Majd, H.; Shariatzadeh, N.; Kalayi, A.; Tayebinejad, N.; Heravifard, S.; Salekzamani, S.; Zahedirad, M. Improvement of vitamin D status via daily intake of fortified yogurt drink either with or without extra calcium ameliorates systemic inflammatory biomarkers, including adipokines, in the subjects with type 2 diabetes. *J Clin Endocrinol Metab* **2012**, *97*, 2005–2011, doi:10.1210/jc.2011-3465.

10. Bonjour, J.P.; Benoit, V.; Payen, F.; Kraenzlin, M. Consumption of yogurts fortified in vitamin D and calcium reduces serum parathyroid hormone and markers of bone resorption: a double-blind randomized controlled trial in institutionalized elderly women. *J Clin Endocrinol Metab* **2013**, *98*, 2915–2921, doi:10.1210/jc.2013-1274.

11. Hower, J.; Knoll, A.; Ritzenthaler, K.L.; Steiner, C.; Berwind, R. Vitamin D fortification of growing up milk prevents decrease of serum 25-hydroxyvitamin D concentrations during winter: a clinical intervention study in Germany. *Eur J Pediatr* **2013**, *172*, 1597–1605, doi:10.1007/s00431-013-2092-6.

12. Khadgawat, R.; Marwaha, R.K.; Garg, M.K.; Ramot, R.; Oberoi, A.K.; Sreenivas, V.; Gahlot, M.; Mehan, N.; Mathur, P.; Gupta, N. Impact of vitamin D fortified milk supplementation on vitamin D status of healthy school children aged 10-14 years. *Osteoporos Int* **2013**, *24*, 2335–2343, doi:10.1007/s00198-013-2306-9.

13. Neyestani, T.R.; Hajifaraji, M.; Omidvar, N.; Nikooyeh, B.; Eshraghian, M.R.; Shariatzadeh, N.; Kalayi, A.; Khalaji, N.; Zahedirad, M.; Abtahi, M.; et al. Calcium-vitamin D-fortified milk is as effective on circulating bone biomarkers as fortified juice and supplement but has less acceptance: a randomised controlled school-based trial. *J Hum Nutr Diet* **2014**, *27*, 606–616, doi:10.1111/jhn.12191.

14. Toxqui, L.; Pérez-Granados, A.M.; Blanco-Rojo, R.; Wright, I.; de la Piedra, C.; Vaquero, M.P. Low iron status as a factor of increased bone resorption and effects of an iron and vitamin D-fortified skimmed milk on bone remodelling in young Spanish women. *Eur J Nutr* **2014**, *53*, 441–448, doi:10.1007/s00394-013-0544-4.

15. Jafari, T.; Faghihimani, E.; Feizi, A.; Iraj, B.; Javanmard, S.H.; Esmaillzadeh, A.; Fallah, A.A.; Askari, G. Effects of vitamin D-fortified low fat yogurt on glycemic status, anthropometric indexes, inflammation, and bone turnover in diabetic postmenopausal women: A randomised controlled clinical trial. *Clin Nutr* **2016**, *35*, 67–76, doi:10.1016/j.clnu.2015.02.014.

16. Kruger, M.C.; Chan, Y.M.; Kuhn-Sherlock, B.; Lau, L.T.; Lau, C.; Chin, Y.S.; Todd, J.M.; Schollum, L.M. Differential effects of calcium- and vitamin D-fortified milk with FOS-inulin compared to regular milk, on bone biomarkers in Chinese pre- and postmenopausal women. *Eur J Nutr* **2016**, *55*, 1911–1921, doi:10.1007/s00394-015-1007-x.

17. Li, Q.; Xing, B. Vitamin D3-Supplemented Yogurt Drink Improves Insulin Resistance and Lipid Profiles in Women with Gestational Diabetes Mellitus: A Randomized Double Blinded Clinical Trial. *Ann Nutr Metab* **2016**, *68*, 285–290, doi:10.1159/000447433.

18. Hajimohammadi, M.; Shab-Bidar, S.; Neyestani, T.R. Consumption of vitamin D-fortified yogurt drink increased leptin and ghrelin levels but reduced leptin to ghrelin ratio in type 2 diabetes patients: a single blind randomized controlled trial. *Eur J Nutr* **2017**, *56*, 2029–2036, doi:10.1007/s00394-017-1397-z.

19. Manios, Y.; Moschonis, G.; Mavrogianni, C.; van den Heuvel, E.; Singh-Povel, C.M.; Kiely, M.; Cashman, K.D. Reduced-fat Gouda-type cheese enriched with vitamin D(3) effectively prevents vitamin D deficiency during winter months in postmenopausal women in Greece. *Eur J Nutr* **2017**, *56*, 2367–2377, doi:10.1007/s00394-016-1277-y.

20. Brett, N.R.; Parks, C.A.; Lavery, P.; Agellon, S.; Vanstone, C.A.; Kaufmann, M.; Jones, G.; Maguire, J.L.; Rauch, F.; Weiler, H.A. Vitamin D status and functional health outcomes in children aged 2-8 y: a 6-mo vitamin D randomized controlled trial. *Am J Clin Nutr* **2018**, *107*, 355–364, doi:10.1093/ajcn/nqx062.

21. Kruger, M.C.; Chan, Y.M.; Lau, L.T.; Lau, C.C.; Chin, Y.S.; Kuhn-Sherlock, B.; Todd, J.M.; Schollum, L.M. Calcium and vitamin D fortified milk reduces bone turnover and improves bone density in postmenopausal women over 1 year. *Eur J Nutr* **2018**, *57*, 2785–2794, doi:10.1007/s00394-017-1544-6.

22. Lovell, A.L.; Davies, P.S.W.; Hill, R.J.; Milne, T.; Matsuyama, M.; Jiang, Y.; Chen, R.X.; Wouldes, T.A.; Heath, A.M.; Grant, C.C.; et al. Compared with Cow Milk, a Growing-Up Milk Increases Vitamin D and Iron Status in Healthy Children at 2 Years of Age: The Growing-Up Milk-Lite (GUMLi) Randomized Controlled Trial. *J Nutr* **2018**, *148*, 1570–1579, doi:10.1093/jn/nxy167.

23. Mohammadi-Sartang, M.; Bellissimo, N.; de Zepetnek, J.O.T.; Brett, N.R.; Mazloomi, S.M.; Fararouie, M.; Bedeltavana, A.; Famouri, M.; Mazloom, Z. The effect of daily fortified yogurt consumption on weight loss in adults with metabolic syndrome: A 10-week randomized controlled trial. *Nutr. Metab. Carbiovasc. Dis.* **2018**, *28*, 565–574, doi:10.1016/j.numecd.2018.03.001.

24. Petrova, D.; Bernabeu Litran, M.A.; Garcia-Marmol, E.; Rodriguez-Rodriguez, M.; Cueto-Martin, B.; Lopez-Huertas, E.; Catena, A.; Fonolla, J. IEffects of fortified milk on cognitive abilities in school-aged children: results from a randomized-controlled trial. *European Journal of Nutrition* **2019**, *58(5)*, 1863–1872.

25. Marwaha, R.K.; Dabas, A.; Puri, S.; Kalaivani, M.; Dabas, V.; Yadav, S.; Dang, A.; Pullakhandam, R.; Gupta, S.; Narang, A. Efficacy of Daily Supplementation of Milk Fortified With Vitamin D2 for Three Months in Healthy School Children: A Randomized Placebo Controlled Trial. *Indian Pediatr* **2021**, *58*, 820–825.

26. Sharifan, P.; Ziaee, A.; Darroudi, S.; Rezaie, M.; Safarian, M.; Eslami, S.; Khadem-Rezaiyan, M.; Tayefi, M.; Mohammadi Bajgiran, M.; Ghazizadeh, H.; et al. Effect of low-fat dairy products fortified with 1500IU nano encapsulated vitamin D(3) on cardiometabolic indicators in adults with abdominal obesity: a total blinded randomized controlled trial. *Curr Med Res Opin* **2021**, *37*, 579–588, doi:10.1080/03007995.2021.1874324.

27. Taghizadeh, N.; Sharifan, P.; Toosi, M.S.E.; Doust, F.N.S.; Darroudi, S.; Afshari, A.; Rezaie, M.; Safarian, M.; Vatanparast, H.; Eslami, S.; et al. The effects of consuming a low-fat yogurt fortified with nano encapsulated vitamin D on serum pro-oxidant-antioxidant balance (PAB) in adults with metabolic syndrome; a randomized control trial. *Diabetes Metab. Syndr.-Clin. Res. Rev.* **2021**, *15*, 7, doi:10.1016/j.dsx.2021.102332.

28. Sharifan, P.; Sahranavard, T.; Rashidmayvan, M.; Darroudi, S.; Fard, M.V.; Mohammadhasani, K.; Mansoori, A.; Eslami, S.; Safarian, M.; Afshari, A.; et al. Effect of dairy products fortified with vitamin d(3) on restless legs syndrome in women with premenstrual syndrome, abdominal obesity and vitamin d deficiency: a pilot study. *BMC Womens Health* **2024**, *24*, 434, doi:10.1186/s12905-024-03159-z.

29. Wagner, D.; Sidhom, G.; Whiting, S.J.; Rousseau, D.; Vieth, R. The Bioavailability of Vitamin D from Fortified Cheeses and Supplements Is Equivalent in Adults12. *The Journal of Nutrition* **2008**, *138*, 1365–1371, doi:https://doi.org/10.1093/jn/138.7.1365.

30. Keane, E.M.; Rochfort, A.; Cox, J.; McGovern, D.; Coakley, D.; Walsh, J.B. Vitamin-D-fortified liquid milk--a highly effective method of vitamin D administration for house-bound and institutionalised elderly. *Gerontology* **1992**, *38*, 280–284, doi:10.1159/000213341.

31. Shab-Bidar, S.; Neyestani, T.; Djazayery, A. Response of central obesity to vitamin D intake in the subjects with type-2 diabetes. *Annals of Nutrition and Metabolism* **2015**, *1)*, 416.

32. Moreira-Lucas, T.S.; Duncan, A.M.; Rabasa-Lhoret, R.; Vieth, R.; Gibbs, A.; Badawi, A.; Wolever, T.M.S. Effect of Vitamin D Fortified Cheese on Oral Glucose Tolerance in Individuals Exhibiting Marginal Vitamin D Status and an Increased Risk for Developing Type 2 Diabetes: A Double-Blind, Randomized Placebo-Controlled Clinical Trial. *Faseb J.* **2016**, *30*, 2.

33. Ganmaa, D.; Stuart, J.J.; Sumberzul, N.; Ninjin, B.; Giovannucci, E.; Kleinman, K.; Holick, M.F.; Willett, W.C.; Frazier, L.A.; Rich-Edwards, J.W. Vitamin D supplementation and growth in urban Mongol school children: Results from two randomized clinical trials. *PLoS One* **2017**, *12*, 13, doi:10.1371/journal.pone.0175237.

34. Nikooyeh, B.; Zahedirad, M.; Kalayi, A.; Shariatzadeh, N.; Hollis, B.W.; Neyestani, T.R. Improvement of vitamin D status through consumption of either fortified food products or supplement pills increased hemoglobin concentration in adult subjects: Analysis of pooled data from two randomized clinical trials. *Nutr Health* **2023**, *29*, 567–574, doi:10.1177/02601060221085351.

35. Sharifan, P.; Darroudi, S.; Rafiee, M.; Geraylow, K.R.; Hemmati, R.; Rashidmayvan, M.; Safarian, M.; Eslami, S.; Vatanparast, H.; Zare-Feizabadi, R.; et al. The effects of low-fat dairy products fortified with 1500 IU vitamin D₃ on serum liver function biomarkers in adults with abdominal obesity: a randomized controlled trial. *J. Heatlh Popul. Nutr.* **2023**, *42*, 11, doi:10.1186/s41043-023-00401-6.
